# Mechanistic Elucidation of Dynamic Deformation Transitions and Energy Absorption in Gyroid Structures Across Relative Densities and Compression Velocities

**DOI:** 10.3390/ma19194171

**Published:** 2026-09-29

**Authors:** Fu Liu, Gaohong Xu, Jiulian Wang, Chong Mei, Xiong Pan, Xiaocheng Li, Chunyu Bai, Xulong Xi

**Affiliations:** 1 School of Intelligent Manufacturing and Control Engineering, Shanghai Polytechnic University, Shanghai 201209, China; 15301410073@163.com (G.X.);; 2 Aircraft Structure and Stress Division, Shanghai Aircraft Design and Research Institute, Shanghai 201210, China; 3 College of Aerospace Engineering, Nanjing University of Aeronautics and Astronautics, Nanjing 210016, China; 4 National Key Laboratory of Strength and Structural Integrity, Aircraft Strength Research Institute of China, Xi’an 710065, China13028512013@163.com (X.X.)

**Keywords:** gyroid, relative density, compression velocity, mechanical response, deformation evolution, specific energy absorption

## Abstract

**Highlights:**

- Highlighted coupled loading rate–density effects on gyroid deformation transitions.
- High-velocity loading increased both initial peak and mean crushing forces.
- Low-density gyroids showed earlier deformation-regime transitions and greater rate sensitivity.
- Numerical simulations predicted SEA convergence with weak density dependence at high-velocity compression.

**Abstract:**

Understanding the rate-dependent deformation and energy absorption behavior of architected lattice structures is essential for developing lightweight impact-resistant materials. This study investigates the effects of loading rate and relative density on the mechanical response of additively manufactured acrylonitrile butadiene styrene (ABS) gyroid structures. Quasi-static and dynamic compression experiments were conducted, and PAM-CRASH finite element simulations were validated against the experimentally investigated compression conditions and subsequently used to explore responses beyond the experimentally accessible velocity range. The results show that both the initial peak crushing force and mean crushing force increase with increasing relative density and loading velocity, with distinct strengthening characteristics from quasi-static loading to high-velocity impact. Increasing loading velocity induces a transition in deformation behavior from homogeneous layer collapse to localized shear banding and fracture. At 100 m/s, the numerical simulations further predict a transition toward inertia-dominated layer-by-layer collapse. Lower-density structures exhibit higher sensitivity to loading rate, with deformation transitions occurring at lower velocities. Although total energy absorption increases with loading velocity, the specific energy absorption shows limited dependence on relative density under high-velocity loading. These results highlight the coupled effects of loading rate and relative density on the deformation and energy absorption of gyroid structures and provide insights into the design of lightweight lattice structures for impact-resistant applications.

## 1. Introduction

Lattice structures are promising candidates for crashworthiness applications in automotive, aerospace, and protective systems owing to their high energy absorption efficiency and low mass [1,2,3,4]. Triply periodic minimal surface (TPMS) structures have attracted growing interest because their mathematically defined geometry allows relative density to be tuned directly for parametric design and optimization [5,6]. Their smooth, continuous, and joint-free surfaces reduce the stress concentrations associated with sharp edges and strut intersections in conventional lattices. At comparable relative densities, some TPMS topologies also exhibit greater membrane-stretching contributions and more distributed deformation than conventional lattices, reducing their susceptibility to localized layer collapse and brittle fracture [7].

The mechanical behavior of TPMS structures is strongly governed by topology, with different topological configurations exhibiting distinct mechanical responses [8]. Representative TPMS topologies include gyroid, primitive, diamond, and I-WP geometries. Previous studies have reported that diamond and gyroid structures exhibit higher plateau stresses under quasi-static compression than other TPMS structures [9]. In particular, the continuous curved geometry of the gyroid and the lack of intersecting planes promote progressive collapse and densification while facilitating a more uniform stress distribution. These characteristics reduce local stress concentrations and contribute to relatively high apparent yield strength, stable stress–strain responses, and favorable energy absorption performance [10]. Further parametric studies have established correlations linking key geometric variables, including cell size [11,12], wall thickness [13,14], and relative density [15,16], to the macroscopic mechanical properties of gyroid structures. As a key factor governing the mechanical performance of TPMS structures, relative density plays an important role in determining their stiffness, compressive strength, and energy absorption capacity [17,18]. Qiu et al. [19] compared TPMS structures with different relative densities under quasi-static compression. Gyroid structures exhibited higher plateau stress and more uniform deformation than I-WP and primitive structures, which were prone to defect-induced localization and X-shaped shear bands at low densities. Increasing the gyroid relative density to 30–40% reduced defect sensitivity and improved deformation stability. Tang et al. [20] performed quasi-static three-point bending tests on 316 L stainless-steel gyroid structures. Increasing relative density from 20% to 50% enhanced their flexural strength and stiffness, while the failure mode shifted from single-sided cracking at 30% to bilateral cracking at higher relative densities. Xu et al. [21] fabricated IN718 gyroid structures with four relative densities ranging from 12.5% to 50% and investigated their quasi-static compression and fatigue loading properties. The experimental results showed that the stiffness, yield strength, and fatigue strength of the gyroid structures were markedly enhanced with increasing relative density. However, increasing relative density generally improves mechanical performance; it may also cause an earlier onset of densification, thereby reducing the energy absorption efficiency [22]. Therefore, various advanced structures, including graded structures [23,24] and hybrid structures [25,26], have been developed to further improve crushing stability and energy absorption capability.

However, previous research on gyroid structures has primarily focused on quasi-static loading, with limited consideration of dynamic effects arising from material strain-rate sensitivity and structural inertia. Existing studies [27,28] conducted at relatively low loading rates indicate that loading rate can affect the strength, energy absorption, and local failure behavior of TPMS structures. However, the reported trends are strongly configuration dependent and remain insufficient to characterize the relationship between loading rate and structural performance [29]. With the growing adoption of lattice structures in aerospace and impact protection applications [30,31,32], practical service conditions increasingly involve high-velocity impacts or blast loading. Previous studies have reported that dynamic loading can markedly enhance crashworthiness indicators, including specific energy absorption (SEA) and yield strength. This enhancement is primarily attributed to the strain-rate sensitivity of constituent materials [33] and inertial effects associated with rapid deformation. In recent years, research on TPMS structures has gradually expanded from quasi-static loading to intermediate and high-strain-rate regimes to investigate the mechanisms governing failure and energy dissipation under extreme loading conditions. AlMahri et al. [34] tested five SS316L sheet-based TPMS topologies fabricated by laser powder bed fusion (L-PBF) under quasi-static compression (0.005 s^−1^) and dynamic compression using a Direct Impact Hopkinson Bar (DIHB) at a strain rate of 2057 s^−1^. All topologies exhibited enhanced mechanical performance under dynamic loading, with the diamond structure achieving the highest SEA, indicating strong potential for high-strain-rate energy absorption. Li et al. [35] investigated Gyroid–IWP hybrid sheet TPMS lattices using quasi-static and DIHB tests supported by finite element simulations. At the strain rate of 2000 s^−1^, the maximum SEA reached 44.26 J/g, exceeding those of uniform gyroid and I-WP lattices by 20.77% and 23.43%, respectively. Generally, the deformation modes of the TPMS structures evolve with increasing impact velocity, transitioning from topology-dominated deformation at low velocities to progressive layer-by-layer collapse once the impact velocity exceeds a critical threshold [36,37]. These dynamic effects substantially alter the stress state and deformation kinematics, indicating that quasi-static models may produce significant errors when used to predict their dynamic response. Yuan et al. [38] conducted quasi-static compression and dynamic impact tests at an impact velocity of 190 m/s; the experimental results showed that gyroid structures transitioned from topology-dominated random layered collapse under quasi-static loading to inertia-dominated layer-by-layer collapse at 190 m/s. Peng et al. [39] combined drop-weight tests with validated numerical simulations to study heat-treated AlSi10Mg gyroids with two relative densities. Higher relative density increased the crushing forces and stabilized the force–displacement response, while the deformation modes were classified as homogeneous, transition, and shock.

In summary, extensive research has examined the mechanical and energy absorption capability of gyroid structures, with relative density receiving particular attention because of its critical influence on structural behavior. However, most previous studies have been limited to quasi-static loading conditions. Although recent investigations have gradually extended to intermediate and high strain rates, they have largely focused on structures with a single relative density. Consequently, the coupled effects of relative density and compression velocity on the dynamic response of gyroid structures remain insufficiently understood. In this study, acrylonitrile butadiene styrene (ABS) was selected as the printing material for gyroid structures due to its high ductility and suitability for additive manufacturing. Compression experiments were combined with finite element simulations to characterize the force response, deformation modes, and energy absorption behavior of ABS gyroid structures. The effects of relative density and compression velocity on their dynamic mechanical responses were subsequently elucidated.

## 2. Materials and Methods

### 2.1. Specimen Design and Fabrication

TPMS structures are generated based on mathematical governing equations. The gyroid surface can be implicitly expressed as follows:

*ϕ_G_* = sin *X* cos *Y* + sin *Y* cos* Z* + sin *Z* cos *X* = *C* (1)

where *X* = 2π*x*/*L*, *Y* = 2π*y*/*L*, *Z* = 2π*z*/*L*; L denotes the unit cell size; *x*, *y*, and *z* are the Cartesian coordinates along three mutually orthogonal axes; and *C* is the level-set constant [40]. The parameter *C* controls the surface offset. In this study, the unit cell size was set to 10 mm, corresponding to a cubic unit cell with dimensions of 10 mm × 10 mm × 10 mm. A 5 × 5 × 5 unit-cell array was adopted, resulting in specimen dimensions of 50 mm × 50 mm × 50 mm. Previous research has shown that increasing the array size beyond 5 × 5 × 5 results in negligible improvements in overall structural performance for gyroid-based TPMS structures [41]. Six relative densities (20%, 25%, 30%, 35%, 40%, and 45%) were considered in this study. The relative density ρ* is defined as follows:

(2) $$\rho *\;=\frac{V_{\mathrm{lattice}}}{V_{\mathrm{solid}}}$$

where *V_lattice_* denotes the volume of solid lattice material generated through isosurface offsetting, and *V_solid_* represents the volume of the cubic design domain defined by the overall specimen dimensions [19]. The target relative densities were achieved by varying the value of *C*. The corresponding *C* values for the six relative densities in the study are listed in Table 1.

**Figure 1:**
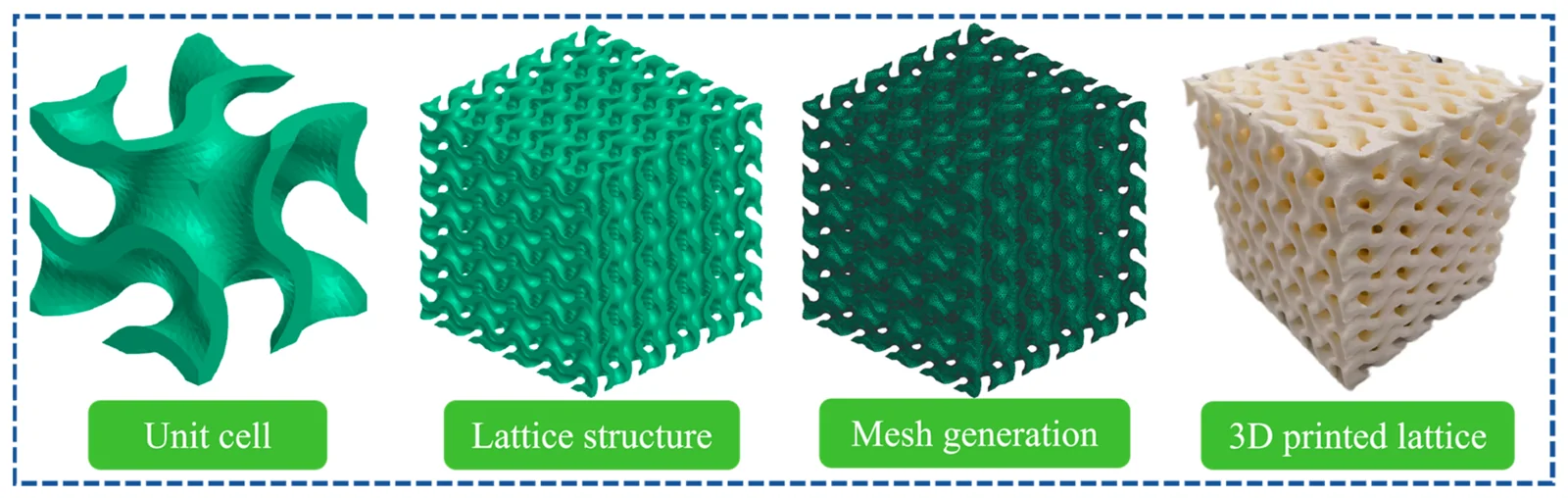
Design and modeling of the gyroid structure.

The unit-cell model was generated using the MSLattice program in MATLAB R2024a and exported in STL format. The model was subsequently imported into HyperMesh 2022, where the unit cell was periodically replicated along three directions to construct the complete lattice structure, followed by mesh generation. The overall modeling procedure is illustrated in Figure 1.

**Figure 2:**
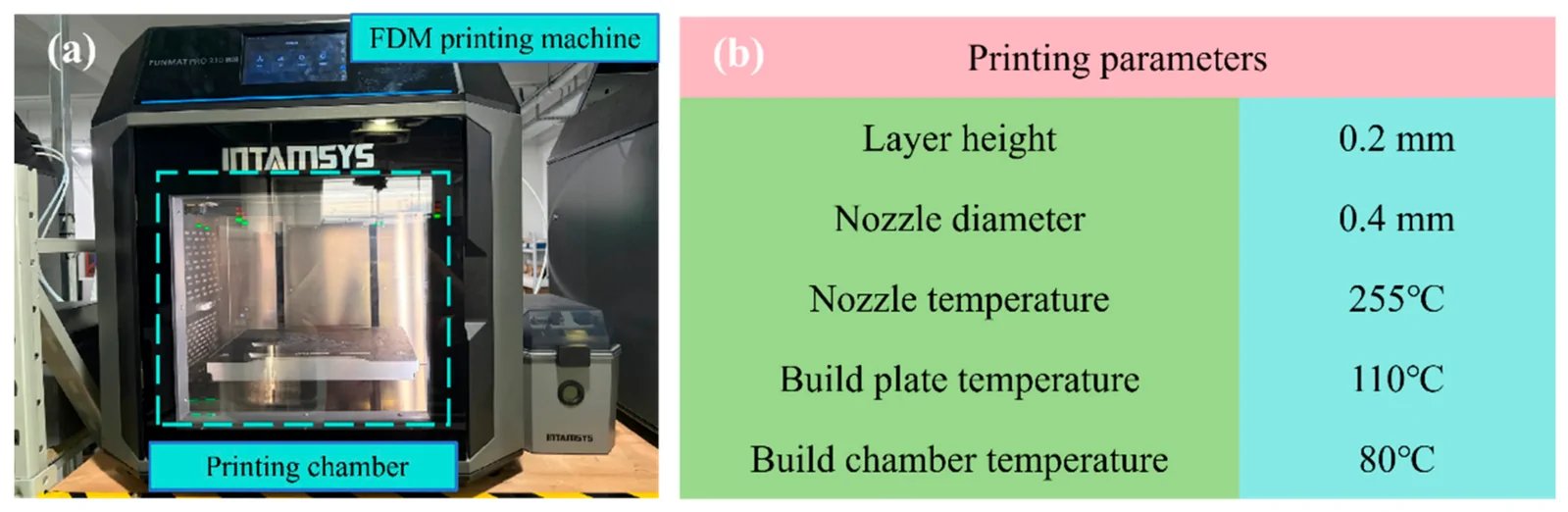
FDM 3D printer and primary printing parameters: (**a**) 3D printing machine, (**b**) printing parameters.

Fused Deposition Modeling (FDM) is considered a suitable additive manufacturing process for fabricating TPMS structures, particularly polymer-based structures [5]. In this study, ABS was used to fabricate tensile coupons for characterizing the mechanical properties of the printed material and gyroid specimens fabricated using equipment provided by Shanghai Chelder Technology Development Co., Ltd. (Shanghai, China) for evaluating their mechanical responses and energy absorption characteristics. All specimens were fabricated using a FUNMAT PRO 310 NEO 3D printer (INTAMSYS Technology Co., Ltd., Shanghai, China), as shown in Figure 2a. The primary printing parameters are summarized in Figure 2b.

### 2.2. Quasi-Static and Dynamic Compression Tests

**Figure 3:**
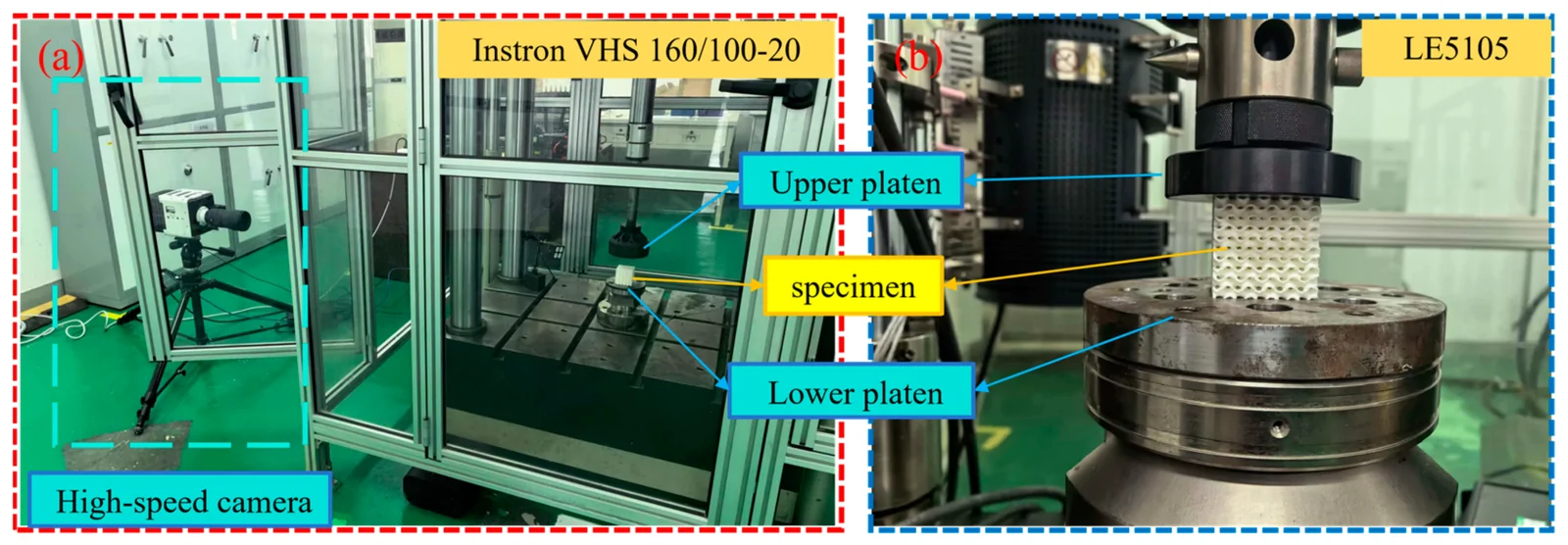
Compression tests of gyroid structures: (**a**) dynamic compression test, (**b**) quasi-static compression test.

Gyroid specimens with relative densities of 30% and 40% were subjected to quasi-static and dynamic compression tests. The dynamic tests were performed using an Instron VHS 160/100–20 high-strain-rate servo-hydraulic testing system (Instron Ltd., High Wycombe, UK), as shown in Figure 3a, whereas the quasi-static tests were conducted using an LE5105 universal testing machine (Lishi (Shanghai) Instruments Co., Ltd., Shanghai, China), as shown in Figure 3b. Each specimen was centered between the upper and lower compression platens. Before loading, the specimen was positioned in contact with the lower platen, after which the upper platen moved downward at a prescribed constant rate. The quasi-static tests were conducted at 6 mm/min, whereas the dynamic tests were performed at 0.1, 1, and 10 m/s. During dynamic compression, a PHANTOM TMX-5010-128G-C high-speed camera (Vision Research, Inc., Wayne, NJ, USA) was used to capture the deformation process. The experimental matrix is summarized in Table 2. The compressive force applied to the specimen was measured using a load cell (Kistler Instrumente AG, Winterthur, Switzerland). Owing to the limited load capacity of the high-strain-rate servo-hydraulic testing system, the maximum nominal compressive strain was restricted to approximately 50%, corresponding to an actuator displacement of 25 mm. This limit was selected with reference to ISO 13314:2011 [42] to prevent equipment overload during densification and to maintain the accuracy of dynamic force measurements. The selected deformation range covered the primary mechanical characteristics of the compressive response, including the initial elastic regime, peak force, and subsequent plateau regime. The experimental compression tests were therefore conducted up to a nominal compressive strain of 50%, which was adopted as the upper deformation limit for the experimental characterization rather than as a criterion for complete densification or energy absorption. To further investigate the post-collapse response and densification behavior beyond the experimentally investigated range, the numerical simulations were extended to a nominal compressive strain of 80%.

### 2.3. Mechanical Response Indicators

**Figure 4:**
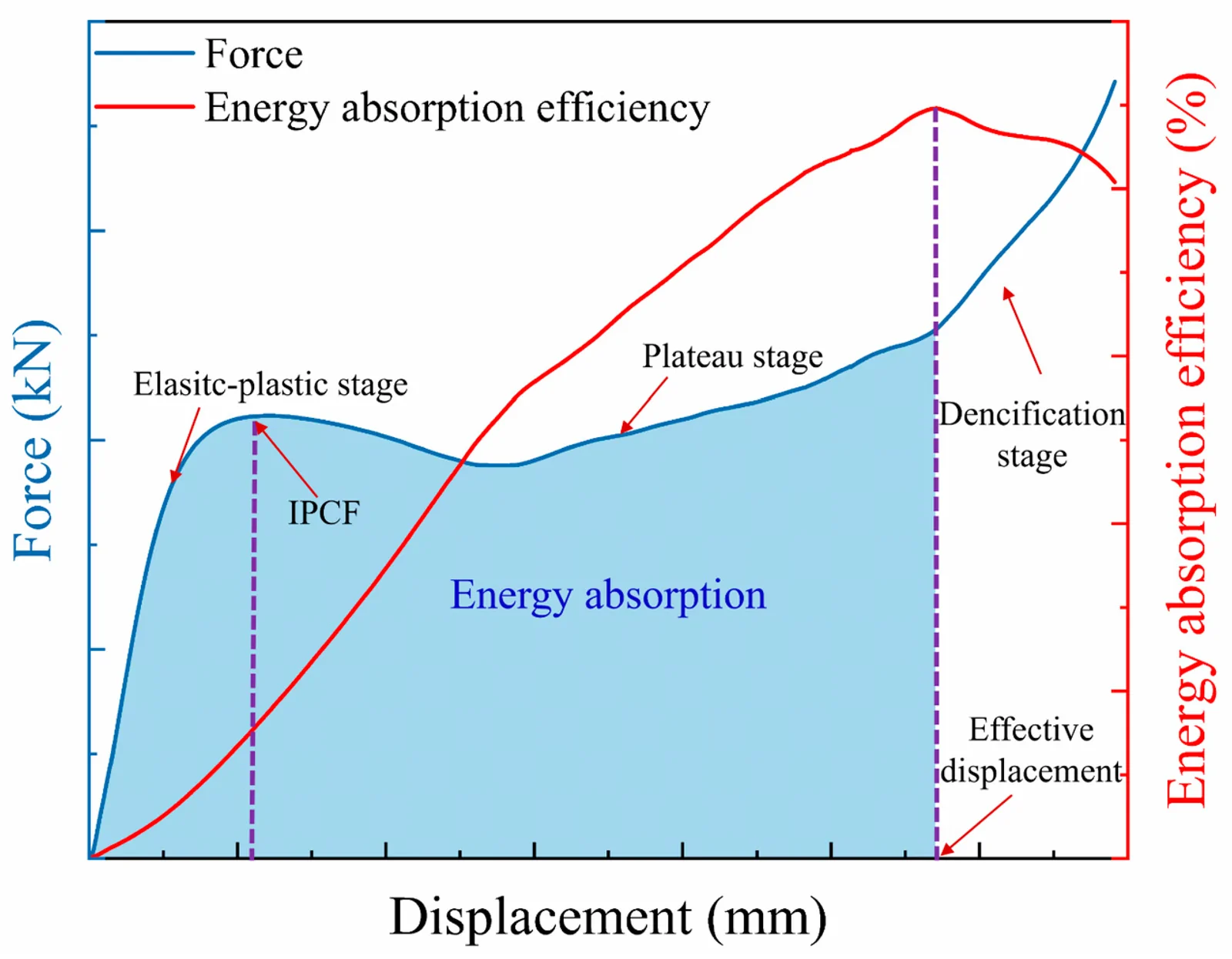
Typical compressive mechanical response of TPMS structures.

Under compressive loading, lattice structures generally exhibit three deformation stages: an initial elastic-plastic stage, a relatively stable force plateau, and a final densification region characterized by a rapid increase in force, as depicted in Figure 4. To comprehensively evaluate the compressive mechanical and energy absorption performance of gyroid structures, several performance indicators were analyzed: energy absorption (EA), specific energy absorption (SEA), initial peak crushing force (IPCF), mean crushing force (MCF), crushing force efficiency (CFE), and energy absorption efficiency.

EA represents the energy absorbed up to a given displacement and corresponds to the area under the force–displacement curve. It is calculated as follows:

(3) $$\mathrm{EA}=\int_{0}^{d}F(x)\mathrm{dx}$$

where *F*(*x*) represents the compressive force at a displacement *x*, and *d* denotes the prescribed or effective crushing displacement used for the corresponding dataset. For the experimental results, *d* was set to 25 mm, corresponding to 50% nominal compressive strain, because the compression tests were terminated at this displacement due to the load capacity of the testing system. For the numerical results, *d* was taken as the effective crushing displacement *d_e_*, which was determined from the complete energy-absorption-efficiency curve.

SEA is defined as the absorbed energy per unit mass and is a critical indicator for assessing the energy absorption capability of lightweight structures. It is expressed as follows:

(4) $$\mathrm{SEA}=\frac{\mathrm{EA}}{M}$$

where *M* is the total mass of the gyroid specimen prior to compression. EA and SEA were evaluated over the experimentally investigated deformation range up to 50% nominal compressive strain. For the numerical simulations, EA and SEA were further evaluated over the extended deformation range up to 80% nominal compressive strain to examine the post-collapse and densification response.

IPCF is defined as the first local maximum of the force–displacement curve and generally represents the transition from the initial elastic-plastic response to structural crushing. An excessively high IPCF indicates a pronounced force spike at the onset of crushing and is therefore undesirable for impact mitigation applications.

MCF represents the average crushing force over the effective crushing displacement and is calculated by dividing the absorbed energy by the effective crushing displacement. It is expressed as follows:

(5) $$\mathrm{MCF}=\frac{\mathrm{EA}}{d}$$

CFE evaluates the ability of a structure to maintain its load-carrying capacity relative to the initial peak force during crushing. A CFE value approaching unity indicates that the mean crushing force is close to the initial peak crushing force, indicating more efficient utilization of the initial peak load. CFE is calculated as follows:

(6) $$\mathrm{CFE}=\frac{\mathrm{MCF}}{\mathrm{IPCF}}$$

Energy absorption efficiency is typically determined from the stress–strain response. Once densification initiates, stress increases rapidly, resulting in a pronounced decrease in energy absorption efficiency. Accordingly, for each force–displacement curve, the effective crushing displacement *d_e_* is determined as the displacement corresponding to the last local maximum of the energy-absorption-efficiency curve before a sustained decrease associated with densification. When multiple local maxima or small oscillations are present, isolated fluctuations that did not mark the onset of sustained efficiency reduction were not taken as the densification point; instead, the final local maximum preceding the subsequent continuous decrease is selected. Since the force–displacement responses were obtained in this study, an equivalent expression based on force and displacement is expressed as follows [43]:

(7) $$\eta (x)=\frac{\int_{0}^{x}F(x)\mathrm{dx}}{F(x)H},\frac{\mathrm{d\eta}(x)}{\mathrm{dx}}{|}_{x=d_{e}}=0$$

### 2.4. Numerical Simulation

#### 2.4.1. Finite Element Modelling

**Figure 5:**
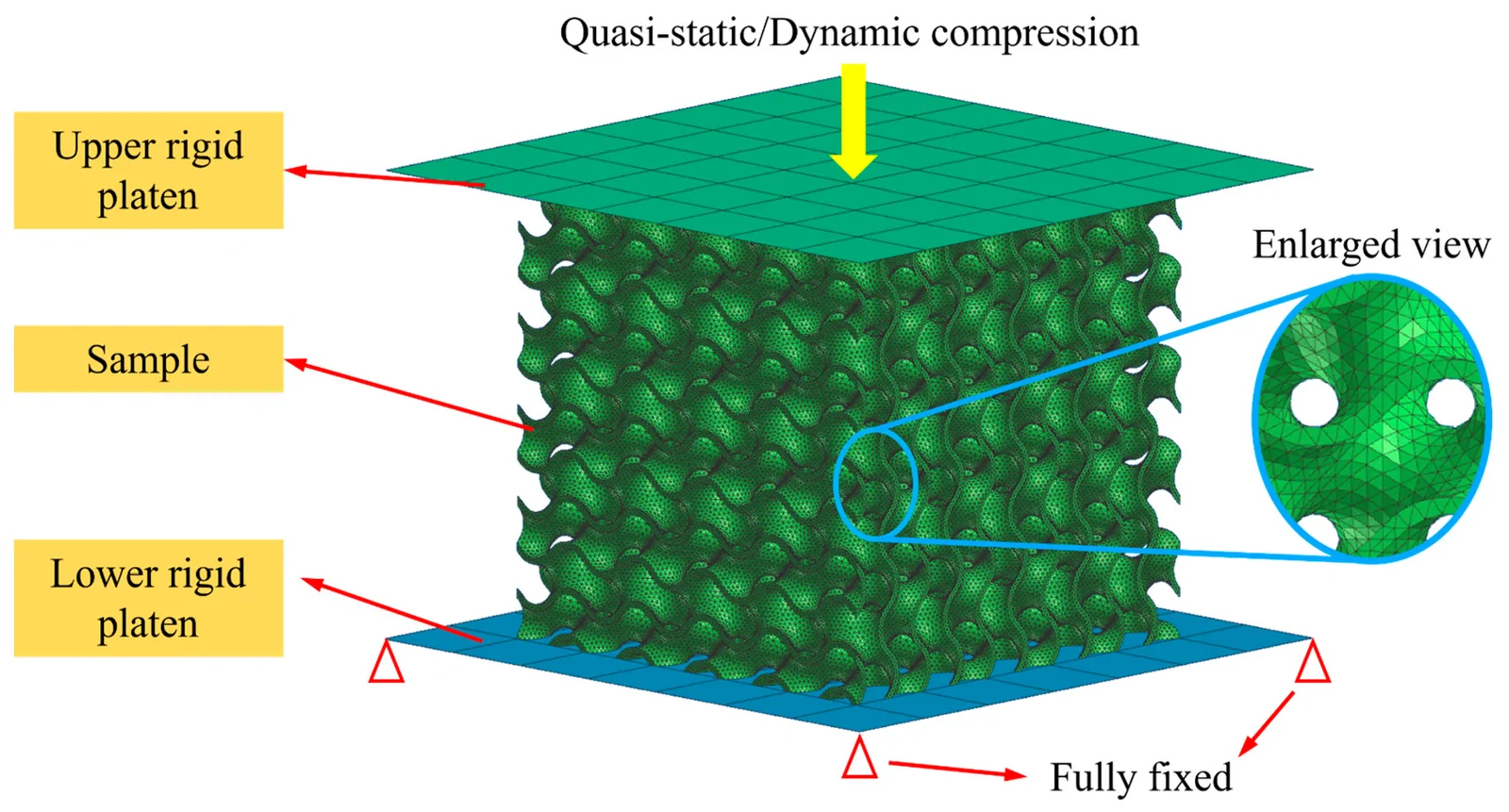
Finite element model of the gyroid structure under compression.

Numerical simulations were performed using PAM-CRASH software 18.0 to investigate the deformation modes, mechanical responses, and energy absorption performance of gyroid structures with different relative densities under various compression velocities. In the finite element model, the upper and lower compression platens used in the experiments were represented by rigid surfaces, as shown in Figure 5. The lower rigid surface was fully constrained, and the gyroid structure was placed on it. A constant prescribed compression velocity was applied to the upper rigid surface along the global z-direction. Contact between the gyroid structure and the two rigid surfaces was modeled using contact Type 33 (Symmetric Node-To-Segment With Edge Treatment) and Type 43 (Edge-To-Edge Contact) to prevent nonphysical interpenetration during compression. Self-contact was also defined for the gyroid structure to account for interactions between cell walls during collapse.

**Figure 6:**
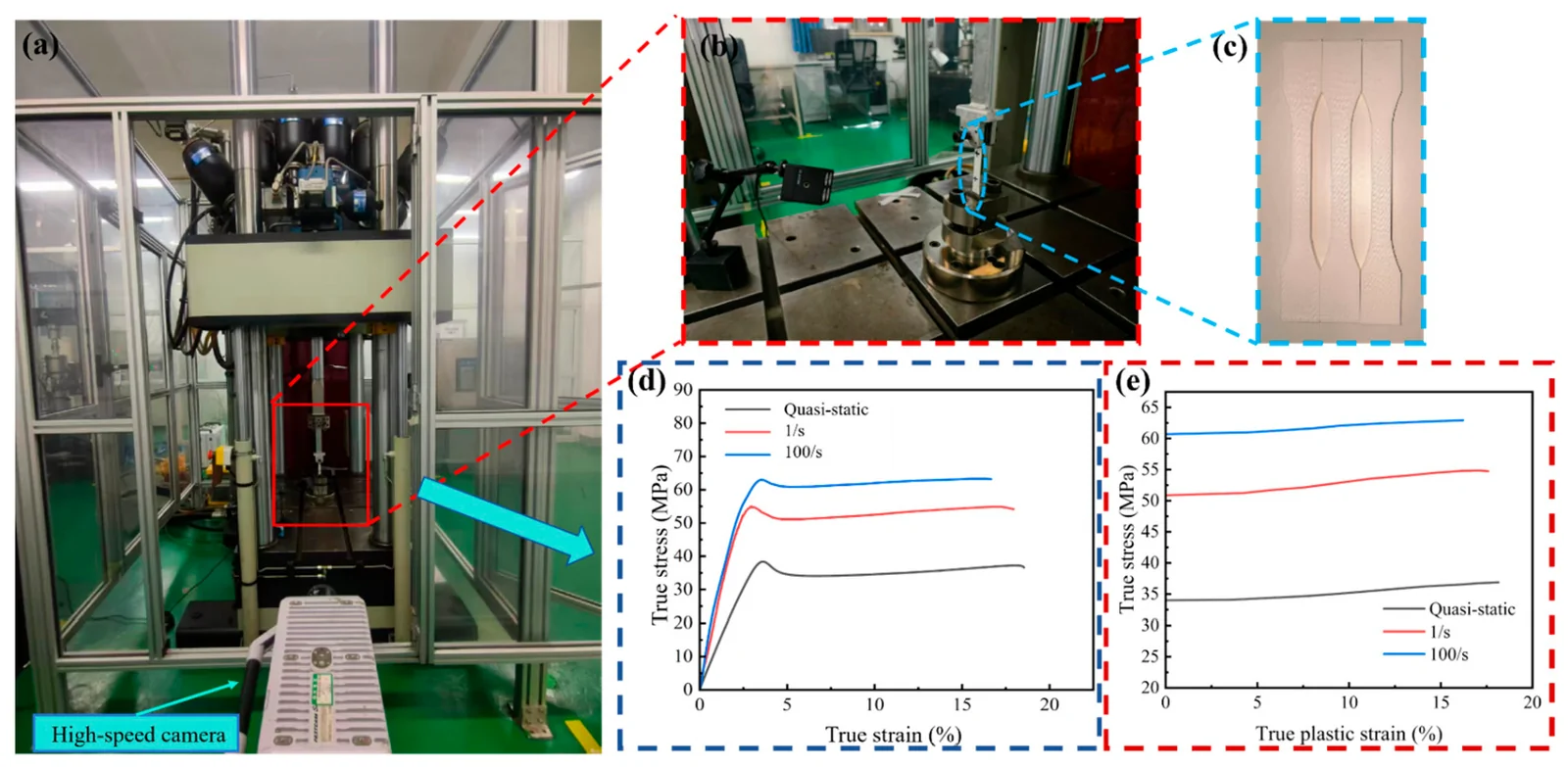
Tensile characterization of ABS: (**a**) dynamic tensile test setup, (**b**) specimen gripping configuration, (**c**) tensile specimen, (**d**) true stress–strain curves of ABS under different strain rates, (**e**) true stress–plastic strain curves of ABS under different strain rates.

ABS was modeled using the MAT 16 (Elastic Plastic with Damage Failure) constitutive model in PAM-CRASH. To characterize its mechanical behavior at different strain rates, quasi-static and dynamic tensile tests were conducted using an LE5105 Static Testing System and an Instron VHS 160/100–20 high-strain-rate servo-hydraulic testing system, respectively, as illustrated in Figure 6a,b. The ABS tensile specimens are shown in Figure 6c. The quasi-static tests were performed at a velocity of 6 mm/min, whereas the dynamic tests were performed at strain rates of 1 and 100 s^−1^. At least three valid specimens were tested under each loading condition. The engineering stress and strain obtained from the tensile tests were converted to true stress and true strain using Equations (8) and (9), respectively.

*ε_true_* = ln(1 + *ε_eng_*) (8)

*σ_true_* = *σ_eng_*(1 + *ε_eng_*) (9)

where *ε_eng_* and *σ_eng_* denote the engineering strain and engineering stress, respectively. The resulting true stress–strain curves are shown in Figure 6d, and the corresponding mechanical properties of ABS are summarized in Table 3. The elastic response in the MAT 16 model was defined using the material density, Young’s modulus, and Poisson’s ratio, while the plastic portions of the experimentally obtained true stress–strain curves at quasi-static, 1, and 100 s^−1^ were used as the corresponding rate-dependent material input curves. The plastic portions (Figure 6e) of the three curves were incorporated into the material, with the corresponding data provided in Table 4, to define the strain-rate-dependent plastic response of the material. A maximum effective plastic strain was introduced as the failure criterion, and this value was calibrated against the experimental compression response, including the damage behavior and force–displacement curves. The initial value of the maximum equivalent plastic strain was set to 0.18, corresponding to the material failure strain obtained from the quasi-static tensile test. The maximum effective plastic strain was then systematically adjusted and calibrated against the experimental IPCF and MCF. Through this calibration procedure, a maximum equivalent plastic strain of 0.4 was determined and adopted as the element failure criterion in the simulations. No progressive damage evolution law was introduced in the present model. Once the prescribed effective plastic strain limit was reached, the corresponding element was considered failed and deleted. The thermal softening effect of the material was not considered in the present study.

#### 2.4.2. Mesh Sensitivity Analysis

**Figure 7:**
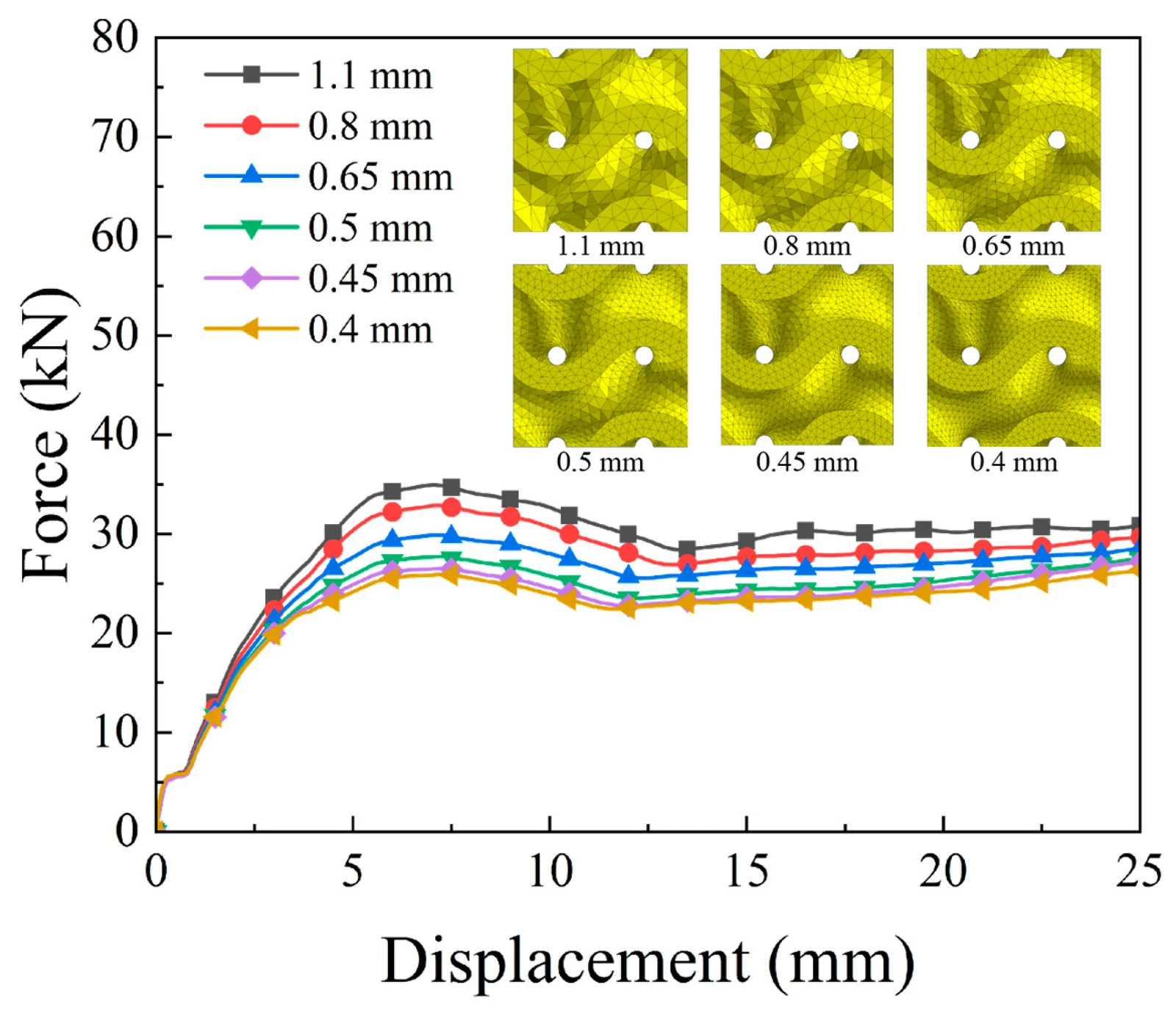
Mesh-sensitivity analysis.

A mesh-sensitivity analysis was performed to assess mesh convergence while balancing numerical accuracy and computational cost. The same gyroid geometry was meshed using different element sizes to evaluate mesh convergence. A gyroid structure with a relative density of 40% was selected, and six element sizes (1.1, 0.8, 0.65, 0.5, 0.45 and 0.4 mm) were examined. The structure was discretized using tetrahedral elements, resulting in 581,059, 847,163, 1,417,428, 2,644,285, 3,683,385, and 4,649,995 elements, respectively. The compression velocity was set to 10 m/s, and the prescribed displacement was 25 mm. Figure 7 compares the simulated force–displacement curves obtained with the different element sizes. The curves obtained with element sizes of 0.5, 0.45, and 0.4 mm exhibited only minor differences, indicating that mesh convergence was achieved within this range. Therefore, an element size of 0.5 mm was selected for the subsequent simulations to balance numerical accuracy and computational cost. Table 5 lists the element counts of the gyroid structures with six relative densities generated using the selected mesh size.

## 3. Results and Discussions

### 3.1. Deformation Modes

**Figure 8:**
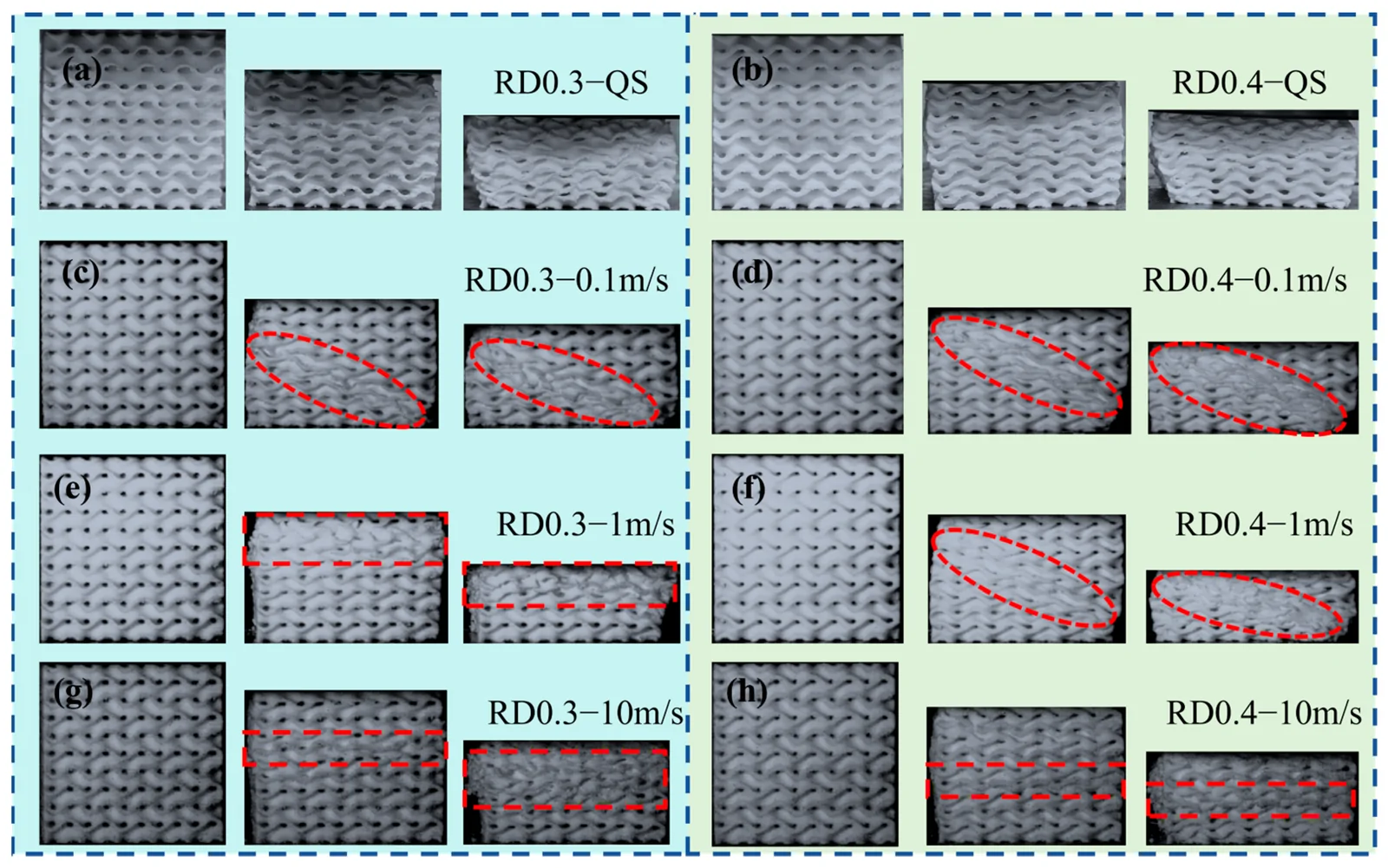
Deformation modes of gyroid structures with two relative densities under different compression velocities: (**a**) RD 0.3–QS, (**b**) RD 0.4–QS, (**c**) RD 0.3–0.1 m/s, (**d**) RD 0.4–0.1 m/s, (**e**) RD 0.3–1 m/s, (**f**) RD 0.4–1 m/s, (**g**) RD 0.3–10 m/s, (**h**) RD 0.4–10 m/s.

The deformation modes of gyroid structures with relative densities of 30% and 40% under different compression velocities are shown in Figure 8. The experimental results demonstrate that compression velocity markedly influenced the deformation mode. Under quasi-static compression, both structures exhibited relatively homogeneous volumetric deformation. As the compression velocity increased, distinct inclined shear bands developed; however, these features disappeared at higher velocities. As shown in Figure 8a,b, the relatively long deformation time under quasi-static compression allowed sufficient load redistribution and coordinated plastic deformation within the gyroid structures. Local yielding and bending of the cell walls could therefore propagate into adjacent regions rather than remaining localized. Consequently, the structures underwent uniform progressive crushing without the formation of distinct shear bands. At a compression velocity of 0.1 m/s, both structures exhibited inclined shear bands during deformation, as shown in Figure 8c,d, representing a characteristic failure mode of gyroid structures [44]. Owing to the pronounced three-dimensional curvature of the continuous gyroid surface, external compressive loads are converted into combined axial forces, bending moments, and shear forces within the cell walls. Once the local stress reaches the yield threshold of ABS, the curved walls begin to deform through bending, followed by yielding or local buckling. Moreover, interlayer bonding interfaces, wall-thickness deviations, and local porosity associated with FDM introduced spatial variations in stiffness and strength, thereby providing preferential sites for deformation localization. With continued loading, localized cell rotation, bending, and collapse propagated approximately along the direction of maximum shear stress, ultimately forming deformation bands extending across multiple cells. Because of the appreciable ductility of ABS at this loading velocity, the localized deformation was manifested primarily as plastic yielding, bending and folding, and relative sliding of the cell walls.

When the compression velocity was further increased to 1 m/s, the gyroid structure with a relative density of 40% exhibited a similar deformation mode, with an inclined shear band and pronounced relative sliding still evident, as shown in Figure 8e. By contrast, the structure with a relative density of 30% underwent a transition to a different deformation mode, as illustrated in Figure 8f. This transition may be consistent with its thinner cell walls, higher wall slenderness, and reduced effective stiffness and crushing strength. Cells near the loading end were initially subjected to higher local compressive stress, bending moments, and contact constraints, causing them to undergo local instability or failure before a continuous inclined shear-localization path could fully develop. Consequently, a horizontal fracture band developed approximately perpendicular to the loading direction. Once formed, this horizontal fracture band disrupted the continuous load-bearing paths within the affected region and served as an initial collapse interface during subsequent crushing. Cells adjacent to the fracture band lost part of their original constraints, causing load redistribution to neighboring intact cell layers. The subsequent deformation then extended outward from the damaged region, ultimately producing a distinct layer-by-layer progressive crushing mode.

When the compression velocity reached 10 m/s, the inclined shear bands disappeared. After the elastic stage, the specimen with a relative density of 30% developed a distinct horizontal fracture band near its mid-height. Layer-by-layer crushing subsequently occurred on both sides of the fracture band until the target compression displacement of 25 mm was reached, as shown in Figure 8g. The specimen with a relative density of 40% exhibited a similar deformation mode, as shown in Figure 8h. This transition was associated with the significantly reduced deformation time scale at the higher compression velocity, which limited the time available for load redistribution and coordinated deformation throughout the structure. The reduced loading time scale also increased the influence of stress-wave effects on load redistribution and deformation coordination, contributing to increasingly non-uniform deformation. Upon completion of the elastic stage, local inertial effects of the cell walls may restrict the coordinated rotation and inclined relative sliding of adjacent cells, thereby suppressing the development and interaction of instability regions along inclined directions. Consequently, deformation became concentrated within a local cell layer approximately perpendicular to the loading direction. Within this layer, the cell walls rapidly accumulate plastic strain and damage under the combined effects of compression, bending, and localized tensile loading. Once the local load-bearing capacity of this layer was exhausted, localized failure propagated laterally across neighboring cells, forming a horizontal localized failure band that subsequently triggered progressive layer-by-layer collapse along the loading direction.

### 3.2. Mechanical Response and Energy Absorption Performance Under Quasi-Static and Dynamic Compression

**Figure 9:**
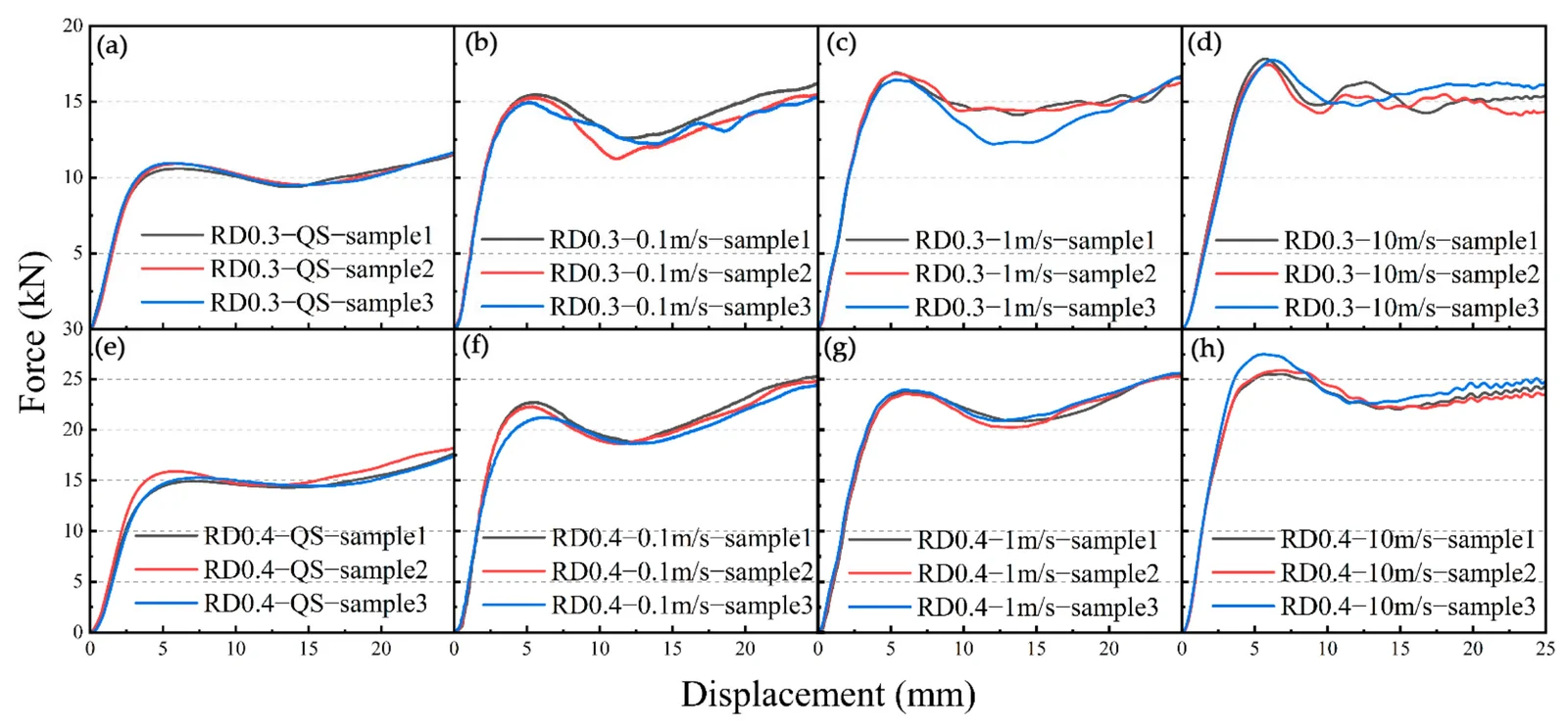
Force–displacement curves of gyroid structures with two relative densities under quasi-static and dynamic compression: (**a**) RD 0.3–QS, (**b**) RD 0.3–0.1 m/s, (**c**) RD 0.3–1 m/s, (**d**) RD 0.3–10 m/s, (**e**) RD 0.4–QS, (**f**) RD 0.4–0.1 m/s, (**g**) RD 0.4–1 m/s, (**h**) RD 0.4–10 m/s.

Figure 9 presents the force–displacement curves of the specimens with relative densities of 30% and 40% under quasi-static and dynamic compression at velocities of 0.1, 1, and 10 m/s. Three repeated tests were conducted under each experimental condition, and the mean values and standard deviations of the main mechanical and energy absorption parameters are summarized in Table 6. Over the displacement range of 0–25 mm, all curves showed an initial elastic regime followed by a crushing plateau. Compared with the quasi-static response, both the IPCF and plateau force were markedly higher under dynamic compression and generally increased with compression velocity, indicating pronounced loading-rate sensitivity. However, as the compression velocity increased from 0.1 to 1 and subsequently to 10 m/s, the increment in plateau force gradually diminished. In particular, the relatively small increase between 0.1 and 1 m/s indicates a weaker dependence of plateau force on compression velocity within this range. Moreover, the force drop from the IPCF to the subsequent plateau increased with compression velocity. The increasing post-peak force drop is likely associated with the combined influence of the strain-rate-dependent material response, structural inertia and the deformation-mode transition. During dynamic compression, strain-rate-dependent strengthening of ABS and the increasing contribution of structural inertia enhance the initial crushing resistance, thereby increasing the IPCF. Once the cell walls begin to yield, the post-yield softening of ABS, together with local buckling, folding, or fracture of the cell walls, leads to a rapid reduction in load-bearing capacity. Increasing compression velocity also reduces the time available for deformation coordination and load redistribution among neighboring cells, contributing to more abrupt and spatially localized instability. The combination of enhanced initial resistance and rapid post-peak degradation of structural stiffness therefore results in a larger force drop.

**Figure 10:**
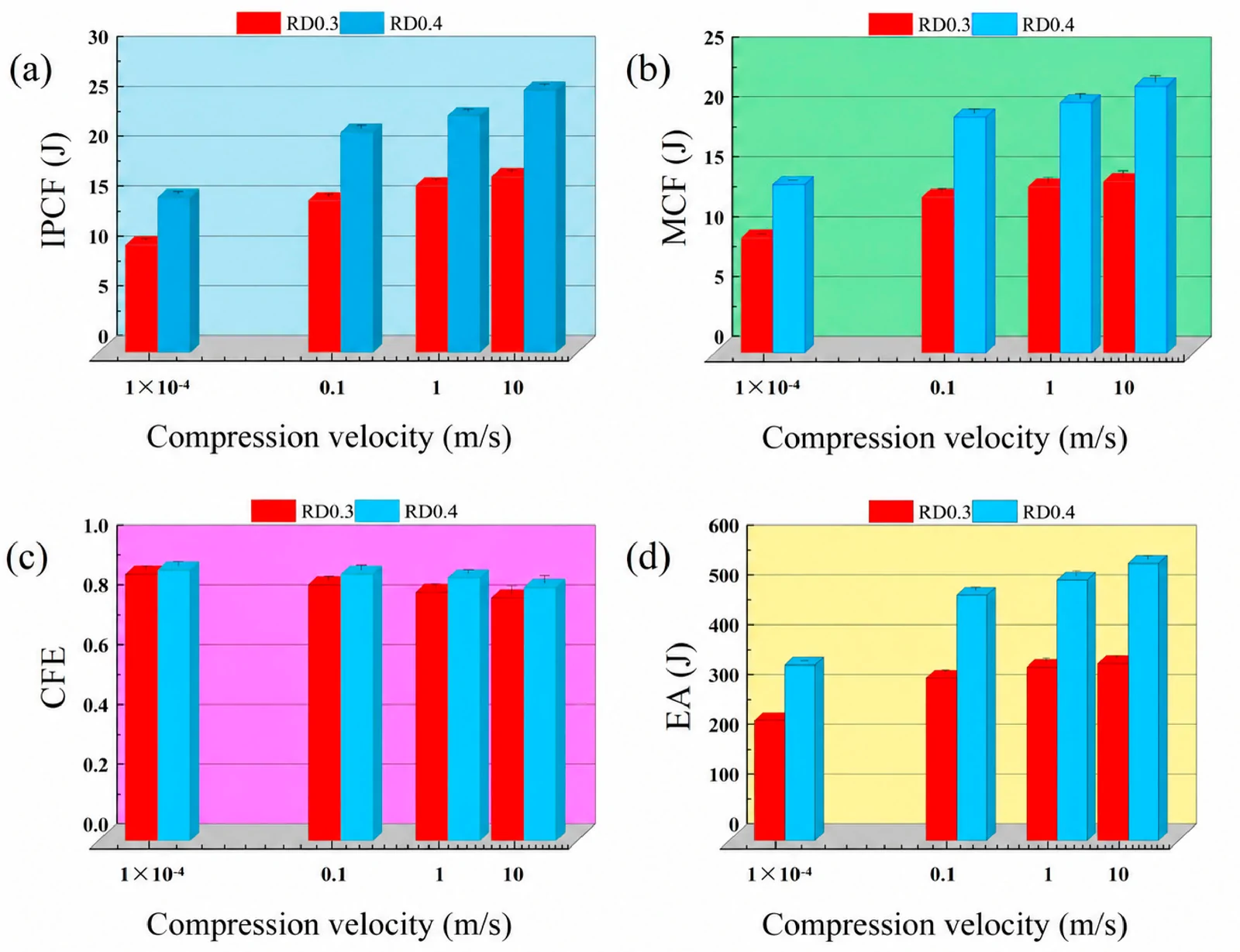
Experimentally measured mechanical performance parameters of gyroid structures with two relative densities: (**a**) IPCF, (**b**) MCF, (**c**) CFE, (**d**) EA.

Figure 10a,b shows the IPCF and MCF, respectively, of gyroid structures with relative densities of 30% and 40% under different compression velocities. For both relative densities, the IPCF and MCF generally increased with compression velocity. This trend can be attributed to the combined effects of strain-rate-dependent material strengthening of ABS and structural inertia within the investigated velocity range. For the structure with a relative density of 40%, both the IPCF and MCF increased steadily with compression velocity. By contrast, for the structure with a relative density of 30%, the increases in both parameters became progressively smaller when the compression velocity exceeded 0.1 m/s. These results indicate that the dynamic mechanical response of gyroid structures is strongly influenced by relative density. In high-relative-density structures, deformation was dominated by post-yield plastic deformation, where the strain-rate-dependent strengthening of the material and pronounced inertial effects act synergistically to substantially enhance the macroscopic load-bearing capacity. In contrast, the deformation of low-relative-density structures was constrained by elastic buckling and brittle failure, making their load-bearing capacity less sensitive to the increase in material yield strength.

Figure 10c,d illustrates the EA and CFE, respectively, of gyroid structures with relative densities of 30% and 40% under different compression velocities. For both structures, EA increased with compression velocity, although the incremental increase gradually diminished from 0.1 to 10 m/s. The reduced growth rate of EA coincided with the velocity-dependent transitions in deformation modes. Although the strain-rate-dependent strengthening of ABS increased the load-bearing capacity, the concurrent development of deformation localization, including inclined shear bands and horizontal failure bands, contributed to structural instability. Such localization limited the extent to which material-level strengthening was reflected in the macroscopic mean crushing force, thereby partially offsetting the benefits of strain-rate strengthening. Consequently, the increments in MCF, and therefore in EA, became progressively smaller over this velocity range. For both relative densities, CFE generally decreased with increasing compression velocity. As the velocity increased, the IPCF increased more rapidly than the MCF, resulting in a reduction in the MCF/IPCF ratio and consequently a lower CFE. These results indicate that increasing compression velocity enhanced the energy absorption over the prescribed displacement but enlarged the disparity between the initial force peak and the sustained crushing force, thereby reducing crushing-force efficiency.

### 3.3. Numerical Model Validation

**Figure 11:**
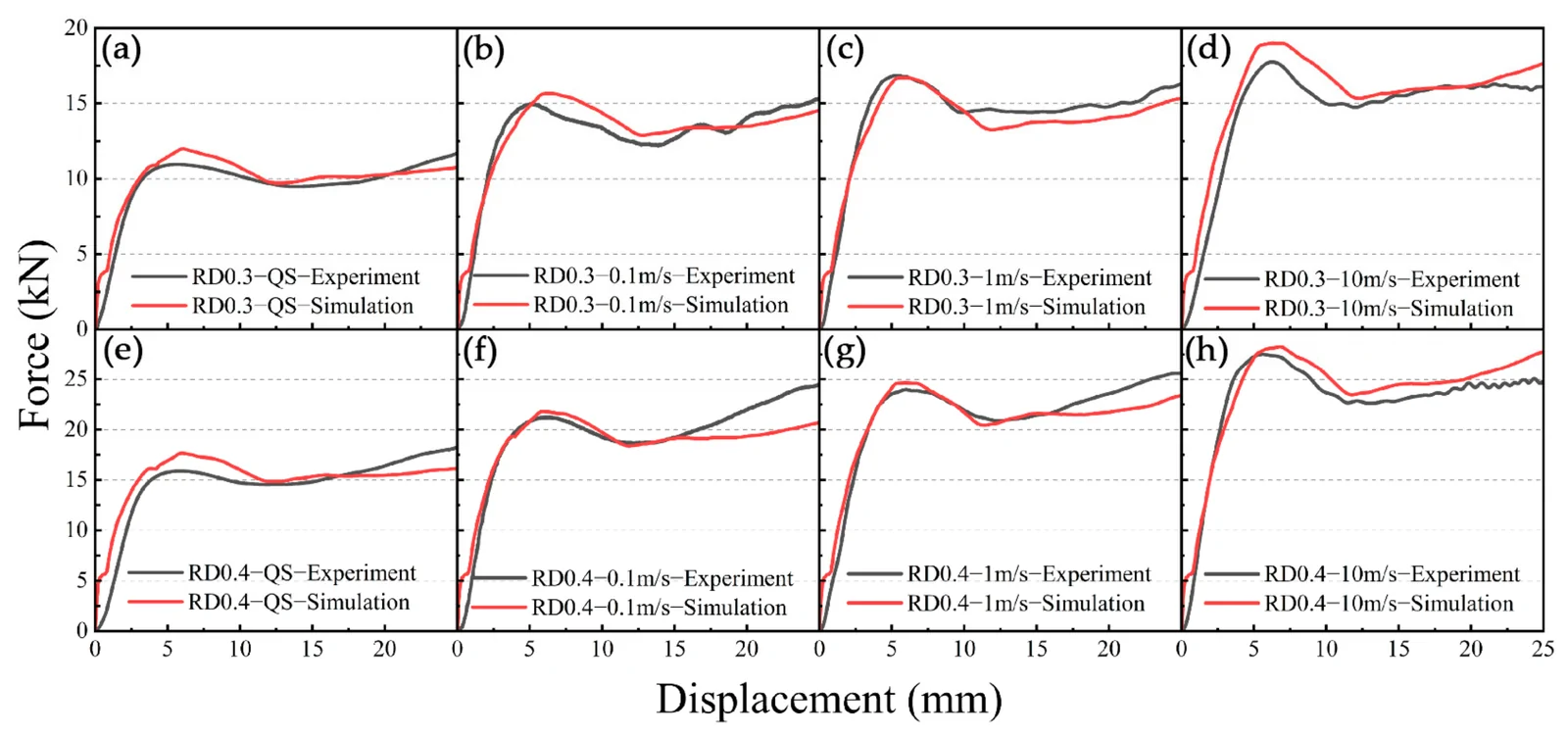
Comparison of force–displacement curves from numerical simulations and experiments: (**a**) RD 0.3–QS, (**b**) RD 0.3–0.1 m/s, (**c**) RD 0.3–1 m/s, (**d**) RD 0.3–10 m/s, (**e**) RD 0.4–QS, (**f**) RD 0.4–0.1 m/s, (**g**) RD 0.4–1 m/s, (**h**) RD 0.4–10 m/s.

**Figure 12:**
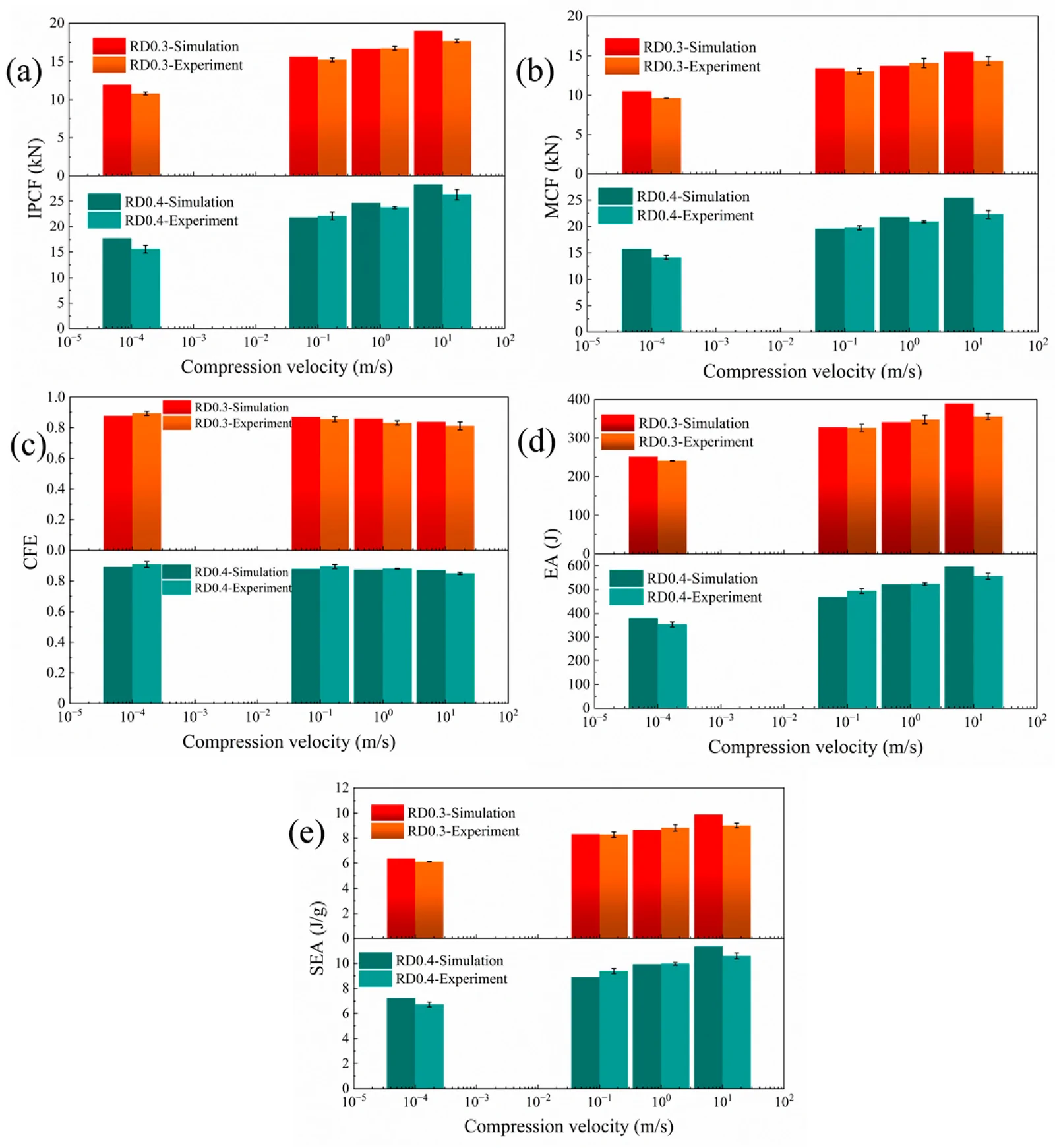
Comparison of experimental and numerical mechanical responses and energy absorption characteristics of gyroid structures with two different relative densities: (**a**) IPCF, (**b**) MCF, (**c**) CFE, (**d**) EA, (**e**) SEA.

Figure 11 compares the numerical and experimental force–displacement curves of specimens with relative densities of 30% and 40% under different compression velocities. The predicted IPCF values agreed closely with the experimental measurements, although the numerical values were consistently slightly higher. This modest overprediction may be attributed, at least partially, to manufacturing imperfections and geometric deviations in the physical specimens that were not represented in the idealized numerical models. During subsequent crushing, the numerical and experimental curves exhibited comparable plateau-force levels. At the onset of loading, the numerical curves exhibited a brief seating stage within the elastic regime. As compression began, some initially inactive contact interfaces were activated abruptly. This transition from open to closed contact states produced transient variations in contact force and effective contact stiffness, appearing as a small step or oscillation in the force–displacement curve. This feature was therefore interpreted as a numerical contact artifact rather than a physical structural response. Despite this initial transient, the model maintained an essentially uniform deformation response during the elastic regime, and the numerical curves remained in good agreement with the experimental results throughout the subsequent post-yield crushing stage. Figure 12 compares the IPCF, MCF, EA, and SEA obtained from the numerical simulations and experiments for structures with relative densities of 30% and 40%. The numerical values of these indicators also showed good agreement with the corresponding experimental measurements.

To quantitatively assess the agreement between the numerical simulations and experimental measurements, the relative errors (absolute error) of the main mechanical and energy absorption parameters were calculated for each experimentally investigated condition. The comparisons of IPCF, MCF, CFE, EA, and SEA are summarized in Table 7, Table 8, Table 9, Table 10 and Table 11, respectively.

**Figure 13:**
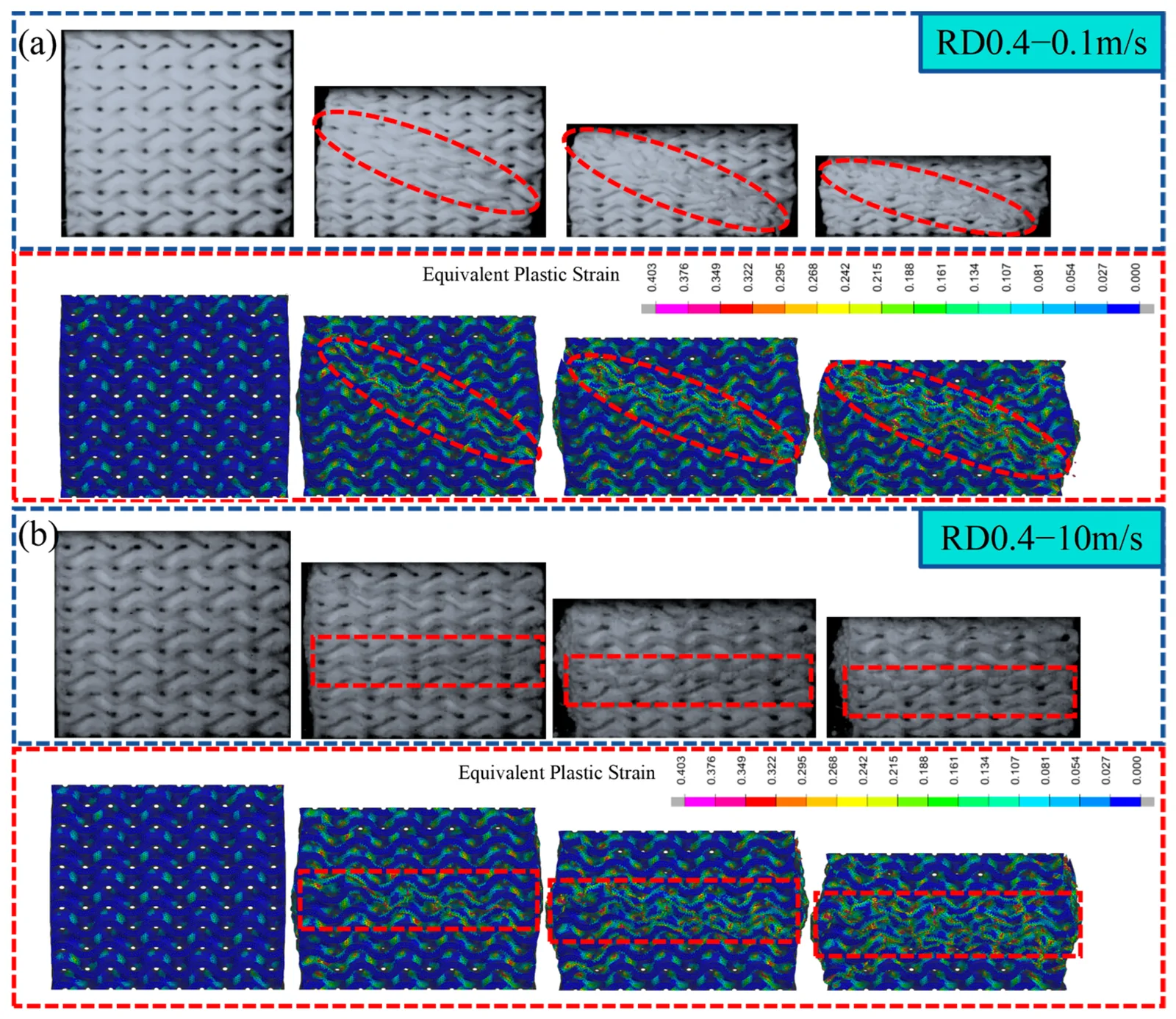
Comparison of experimental and simulated deformation modes: (**a**) relative density of 40% under a compression velocity of 0.1 m/s, (**b**) relative density of 40% under a compression velocity of 10 m/s.

Figure 13 compares the experimental and simulated deformation sequences of the gyroid structure with a relative density of 40% under compression velocities of 0.1 m/s and 10 m/s. The numerical model reproduced the experimentally observed change in the orientation of the dominant deformation-localization band. At 0.1 m/s, the model predicted an inclined localization band consistent with the experimentally observed shear band, whereas at 10 m/s, the predicted localization band was approximately horizontal and was followed by layer-by-layer collapse along the loading direction, consistent with the experimentally observed deformation pattern. Together with the agreement in the force response described above, these comparisons demonstrate that the model can reproduce the global crushing behavior and the principal deformation-mode transition within the experimentally investigated velocity range. The mechanisms underlying these deformation modes are discussed in detail in Section 3.1.

## 4. Parametric Numerical Study

### 4.1. Effect of Relative Density on Mechanical Responses and Energy Absorption

To further investigate the effects of relative density on the mechanical response and energy absorption performance of gyroid structures over an extended crushing range, a parametric numerical study was conducted using the validated numerical framework. The simulations were performed for structures with relative densities of 20%, 25%, 30%, 35%, 40%, and 45%. Four compression velocities were considered: 6 mm/min (0.0001 m/s), 0.1 m/s, 1 m/s, and 10 m/s. These numerical cases extend the relative-density range beyond the experimentally investigated 30% and 40% structures. Therefore, the experimental validation of the coupled effects of relative density and compression velocity is limited to the 30% and 40% structures, while the broader parametric trends are based on numerical predictions. The prescribed compression displacement was set to 40 mm, corresponding to a nominal compressive strain of 80%.

**Figure 14:**
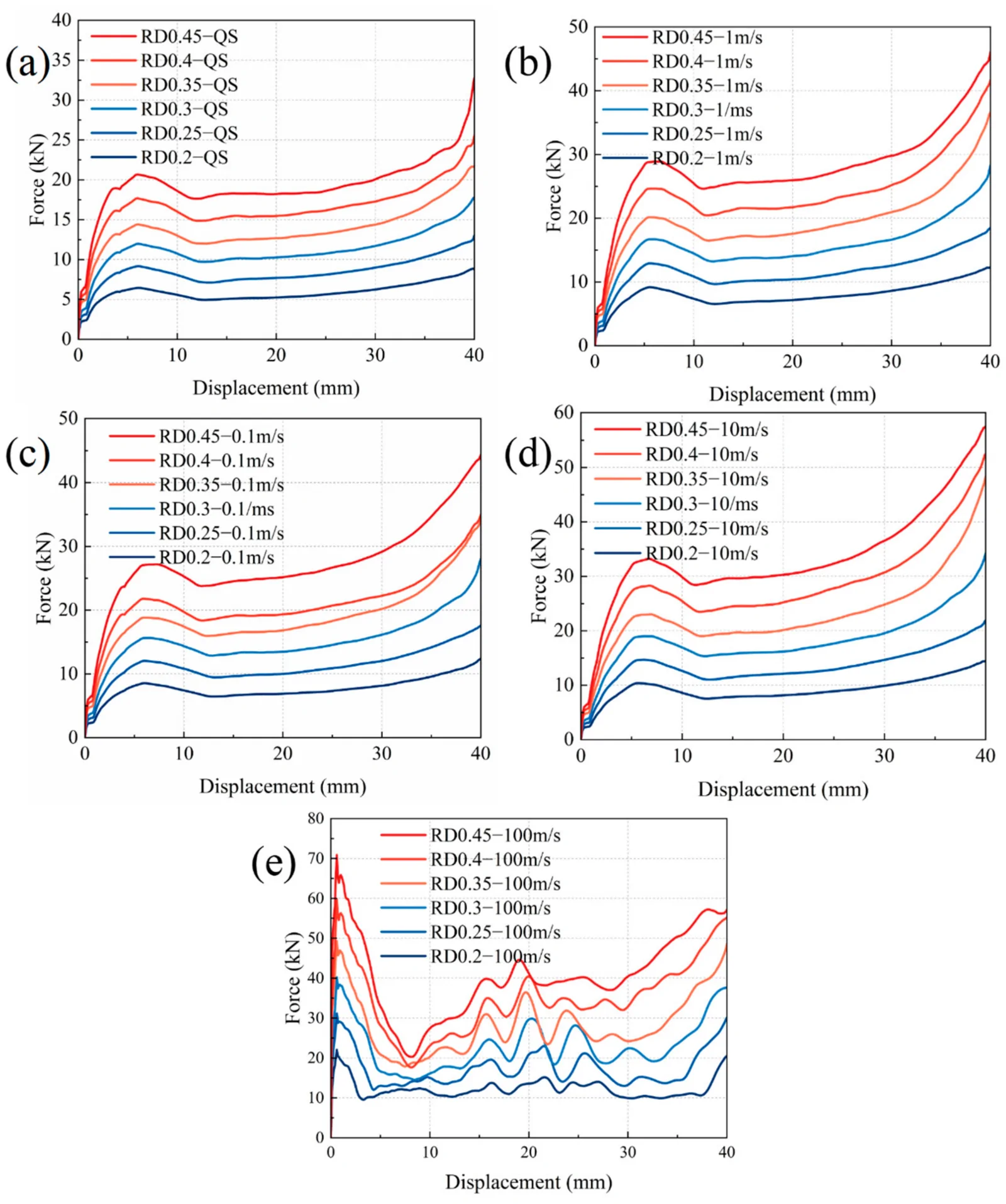
Force–displacement curves of gyroid structures with relative densities ranging from 20% to 45% under different compression velocities: (**a**) 6 mm/min (quasi-static, QS), (**b**) 0.1 m/s, (**c**) 1 m/s, (**d**) 10 m/s, (**e**) 100 m/s.

Figure 14 presents the simulated force–displacement curves of gyroid structures with different relative densities at the four compression velocities. At each velocity, the overall compressive force level increased with relative density. These results demonstrate that relative density has a pronounced influence on the load-bearing response of gyroid structures under both quasi-static and dynamic compression.

### 4.2. Effect of Compression Velocity on Mechanical Responses and Energy Absorption

**Figure 15:**
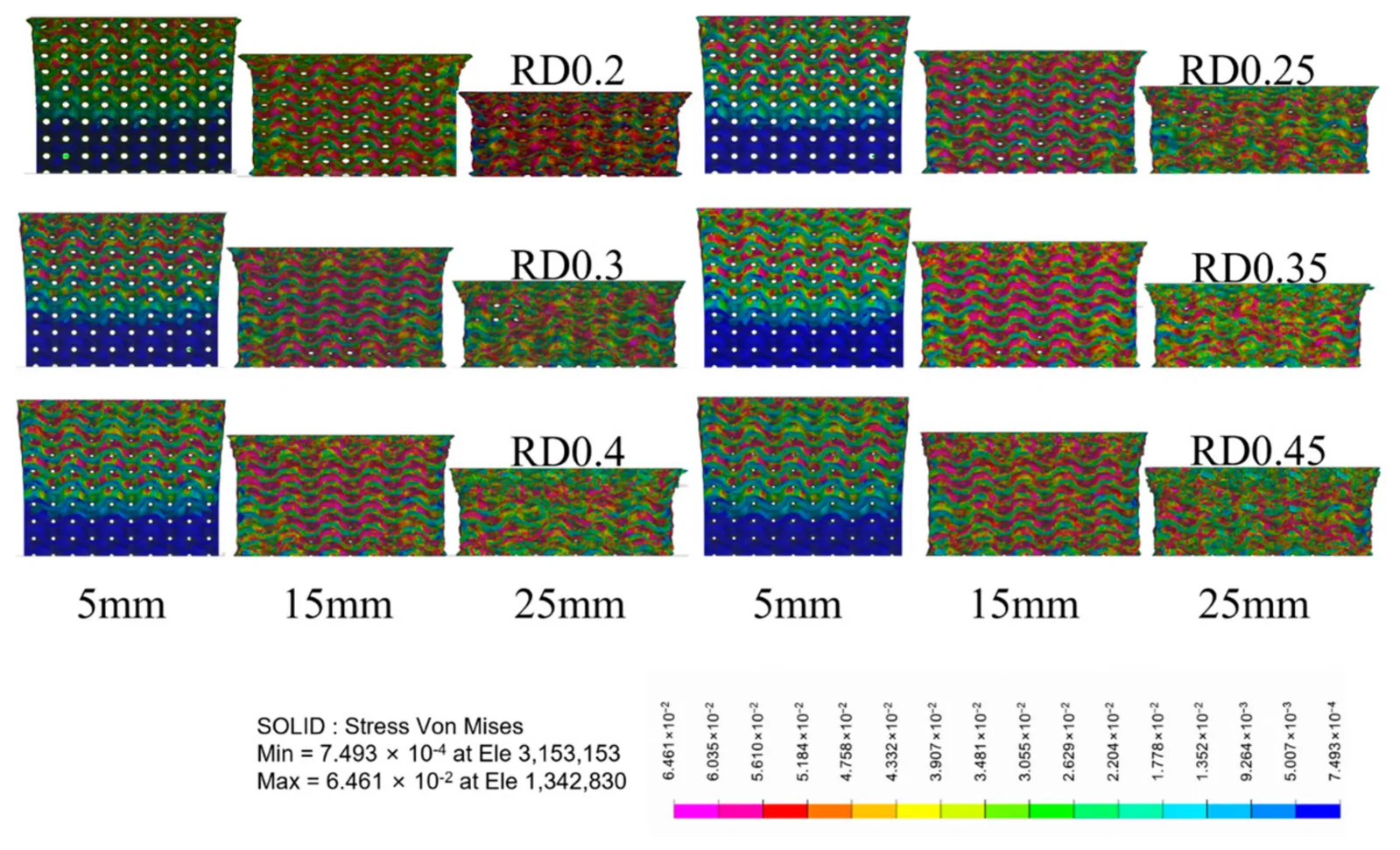
Simulated deformation sequences of gyroid structures at a compression velocity at 100 m/s.

To further examine the dynamic response of ABS gyroid structures at a higher compression velocity, additional simulations were performed at 100 m/s for structures with relative densities ranging from 20% to 45%. Since 100 m/s lies outside the experimentally validated velocity range, the corresponding results are interpreted primarily as numerical predictions of comparative trends rather than as fully validated quantitative responses. Figure 15 presents the corresponding simulated deformation sequences. The predicted deformation patterns indicate an increased influence of structural inertia, with all structures exhibiting layer-by-layer collapse that progresses from the loading end toward the support end.

Figure 14e presents the simulated force–displacement curves of the gyroid structures with relative densities ranging from 20% to 45% under a compression velocity of 100 m/s. The curves exhibited characteristic high-velocity crushing behavior and could be divided into four regimes: a brief elastic regime, an initial force peak followed by a rapid force drop, a strongly oscillatory dynamic crushing plateau, and a densification regime. At the onset of compression, the compressive disturbance propagated from the loading end into the structure as a stress wave. The simulations suggest that the force reached its initial peak before dynamic stress equilibrium was established throughout the specimen. Immediately after this peak, the force decreased sharply. During this interval, the stress wave had not yet fully reached the support end, whereas cell walls within the local high-strain region near the loading end had already undergone concentrated buckling, folding, damage, or fracture, thereby triggering element deletion. The resulting loss of local stability degraded the continuous load-bearing paths, whereas the release of stored elastic strain energy contributed to the pronounced post-peak force drop. At 100 m/s, the limited time available for load redistribution led to the failure of several neighboring cells over a short interval, resulting in more abrupt post-peak drops than those observed at lower velocities. As the local load-bearing paths progressively degraded, the force continued to decrease until a new dynamic crushing state developed, marking the onset of the oscillatory plateau regime. Notably, the initial peak force, post-peak minimum force, and absolute peak-to-minimum force drop all increased with relative density. For the gyroid structure with a relative density of 45%, the force decreased from 70.9 to 20.3 kN, corresponding to an absolute reduction of 50.6 kN. By comparison, the absolute force drop for the structure with the lowest relative density of 20% was only 12.1 kN. The compression displacement corresponding to the post-peak valley also increased with relative density. This trend is consistent with the observation that structures with higher relative densities underwent greater compression before the initial localized crushing region lost its load-bearing capacity and the subsequent dynamic crushing regime developed. Structures with higher relative densities contain thicker cell walls and more robust continuous load-bearing paths, which are associated with greater initial stiffness, dynamic load-bearing capacity, and effective structural impedance. Before the initial peak was reached, more cell-wall material participated in load carrying and therefore stored greater elastic strain energy. Once a horizontal localized failure band or initial crushing layer formed, multiple load-bearing paths within this region underwent concentrated buckling, damage, or fracture over a short interval. The resulting rapid degradation of overall structural stiffness, together with the release of stored elastic strain energy, contributed to the larger absolute peak-to-minimum force drop.

The structures subsequently entered the dynamic crushing plateau regime. Unlike the relatively smooth plateau responses observed under quasi-static and low-to-intermediate-velocity compression, the force–displacement curves at 100 m/s exhibited pronounced serrations with large amplitudes. These force oscillations did not arise solely from layer-by-layer collapse; cell-wall inertia, intermittent contact and compaction, and stress-wave propagation also contributed to their magnitude and temporal evolution. At lower loading velocities, more time was available for stress redistribution throughout the structure. Buckling and fracture events in different cell regions were more temporally dispersed and could overlap, causing their individual contributions to be averaged in the global force response and producing a relatively smooth plateau. Under high-velocity loading, the local inertia of the cell walls restricted coordinated deformation among different regions, causing the force and deformation to become concentrated within a limited number of cell layers. Once these layers reached their failure threshold, relatively synchronous buckling or fracture could cause a rapid loss of load-bearing capacity and generate a local force minimum. Subsequent contact and compaction within the crushed region, together with the renewed load bearing of the adjacent intact cell layers, increased the force and produced the next peak. Meanwhile, stress-wave propagation and reflection within the structure further altered the reaction force measured at the loading end. The large-amplitude oscillations in the high-velocity plateau can therefore be interpreted as the macroscopic manifestation of the coupled effects from dynamically localized layer-by-layer crushing, cell-wall inertia, intermittent contact and compaction, and stress-wave propagation.

During the final stage of compression, a large proportion of the internal voids gradually closed, and extensive contact developed among the damaged or folded cell walls, substantially reducing the remaining compressible space. The applied force was then used primarily to further deform and compact the contacting ABS material and to overcome the increasing contact and frictional resistance between the cell walls. Consequently, the force increased rapidly with displacement, marking the onset of densification.

**Figure 16:**
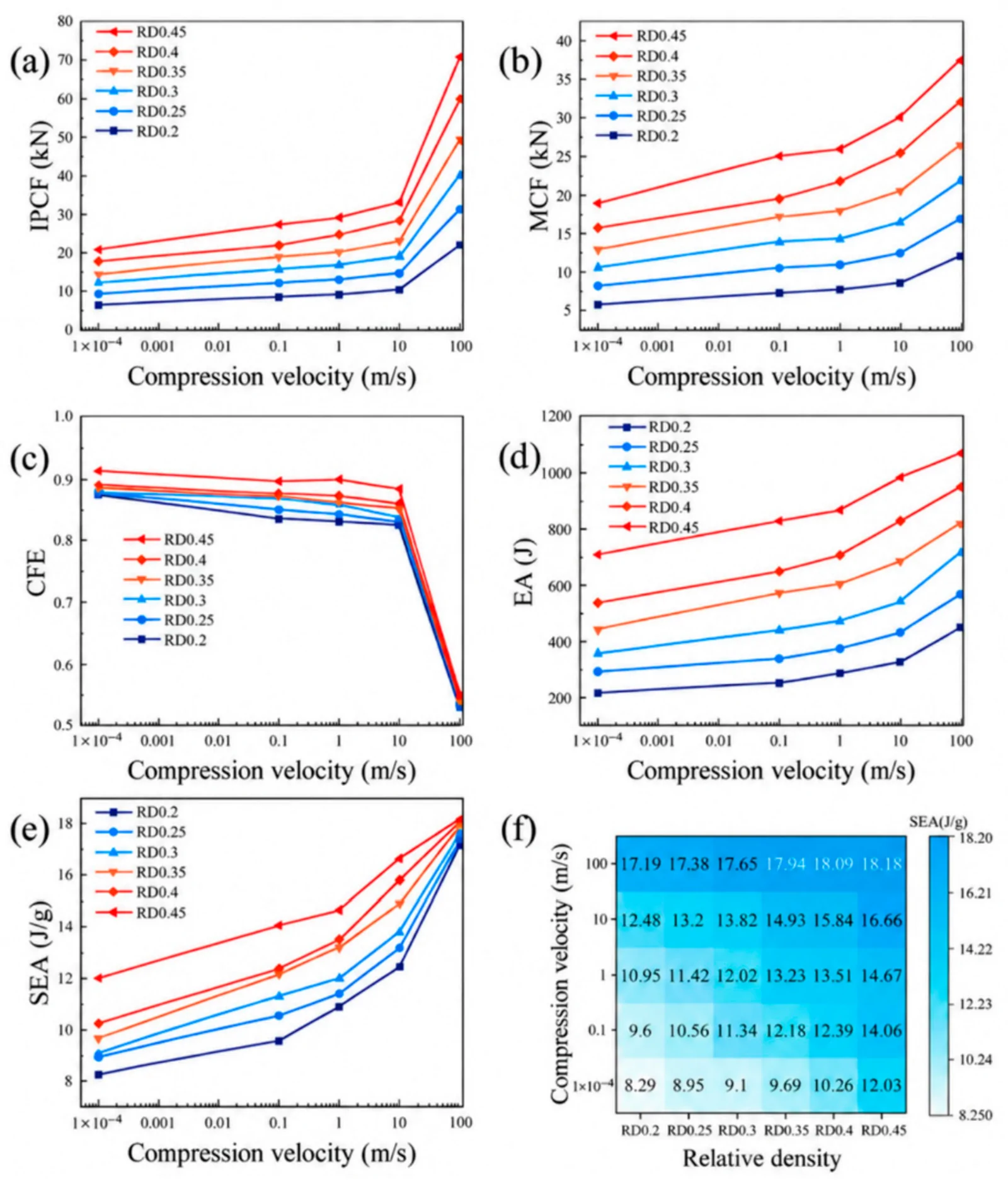
Numerically predicted dynamic response parameters of gyroid structures with different relative densities: (**a**) IPCF, (**b**) MCF, (**c**) CFE, (**d**) EA, (**e**) SEA, (**f**) SEA heatmap.

The performance indicators for all simulated configurations are compared in Figure 16. Overall, the IPCF (Figure 16a) and MCF (Figure 16b) of the gyroid structures increased with both compression velocity and relative density. The increase with compression velocity is consistent with the rate-dependent constitutive response of ABS and the increasing contribution of structural inertia, whereas the increase with relative density results from thicker cell walls and more effective load-bearing paths. Together, these factors enhance the absolute load-bearing capacity of the structures. The IPCF and MCF increased comparatively gradually from 6 mm/min and 10 m/s but rose sharply when the compression velocity reached 100 m/s. For the structure with a relative density of 45%, the IPCF increased from 33.22 kN at 10 m/s to 70.85 kN at 100 m/s. The increase in crushing strength with compression velocity is attributed to the combined effects of the strain-rate sensitivity of ABS and structural dynamic effects. Their individual contributions were not quantitatively separated in the present study. CFE remained relatively stable at approximately 0.80–0.90 between 6 mm/min and 10 m/s but decreased sharply at 100 m/s, as shown in Figure 16c. At this velocity, the IPCF increased much more rapidly than the MCF, substantially widening the disparity between the initial force peak and the sustained mean crushing force. The reduction in CFE therefore indicates a deterioration in crushing-force efficiency and an increased initial-peak penalty. Although CFE did not directly quantify force fluctuations, its sharp decrease, together with the pronounced serrations in the plateau response, suggested a less stable load-bearing response at 100 m/s. Both EA and SEA increased with increasing compression velocity, as shown in Figure 16d,e, respectively. Figure 16f presents a heat map of SEA as a function of relative density and compression speed. SEA generally increased with both relative density and compression velocity. The dependence of SEA on relative density weakened as the compression velocity increased. From quasi-static loading to intermediate compression velocities, the SEA retained a clear relative-density-dependent hierarchy, with denser structures providing greater energy absorption per unit mass. At 100 m/s, however, the SEA values of the different structures converged to a narrow range of 17.19 to 18.18 J/g, and the difference between the high-density structures (e.g., RD = 0.45) and low-density structures (e.g., RD = 0.20) was markedly reduced. At 100 m/s, the larger post-peak force drops observed in structures with higher relative densities reduced their sustained load-bearing advantage over a substantial displacement range. Consequently, the increase in MCF was smaller than that in IPCF. Although the denser structures withstood higher instantaneous forces, this advantage was not maintained throughout the dynamic crushing plateau. They also reached densification at smaller crushing displacements, shortening the effective plateau stroke available for energy absorption. By contrast, structures with lower relative densities exhibited lower peak and plateau forces but had smaller masses and longer effective crushing strokes. Thus, the higher absolute forces sustained by the denser structures were offset by their larger post-peak force drops, shorter effective plateau strokes, and greater masses.

## 5. Conclusions

ABS gyroid structures were fabricated by FDM, and their mechanical responses and energy absorption characteristics under quasi-static and dynamic compression were investigated through a combination of experiments and numerical simulations. The principal conclusions are as follows:

1. Within the experimentally investigated loading range from quasi-static compression to 10 m/s and at nominal compressive strains of up to 50%, the developed finite element model reproduced the measured force–displacement responses. The predicted IPCF, MCF, CFE, EA, and SEA agreed with the corresponding experimental results. The model also captured the experimentally observed transitions in deformation mode with increasing compression velocity.

2. Compression velocity had a pronounced effect on the deformation mode. Under quasi-static compression, the structures exhibited relatively homogeneous volumetric deformation. At 0.1 m/s, inclined shear bands developed in the structures with relative densities of both 30 and 40%. When the compression velocity increased to 1 m/s, the 40% relative-density structure retained the inclined shear-band mode, whereas the 30% relative-density structure transitioned to horizontal fracture followed by layer-by-layer collapse. At 10 m/s, inclined shear-band localization was no longer observed, and horizontal localized failure bands formed approximately perpendicular to the loading direction. At 100 m/s, the simulations predicted pronounced layer-by-layer crushing from the loading end toward the support end, associated with stronger local inertial effects and asynchronous load transmission. This behavior was consistent with the increased influence of cell-wall inertia and the insufficient time available for load redistribution and equilibration throughout the structure.

3. The gyroid structures exhibited clear loading-rate sensitivity. The IPCF and MCF generally increased with both compression velocity and relative density. Their increases with compression velocity were consistent with the rate-dependent strengthening of ABS and the increasing contribution of structural inertia, whereas their increases with relative density resulted from thicker cell walls and more effective load-bearing paths. At 100 m/s, the numerically predicted IPCF increased much more sharply than the MCF. The resulting reduction in CFE, together with the large-amplitude serrations in the dynamic crushing plateau, indicated reduced crushing-force efficiency and a less stable load-bearing response.

4. EA generally increased with compression velocity and relative density over the investigated conditions. At 100 m/s, however, the predicted SEA values of structures with relative densities ranging from 20% to 45% converged to a narrow range of 17.19–18.18 J/g. Although structures with higher relative densities sustained greater instantaneous forces, their pronounced post-peak force reductions limited the persistence of this load-bearing advantage. They also reached densification at smaller crushing displacements, shortening the effective plateau stroke. Together with their greater masses, these effects weakened their energy absorption advantage per unit mass. Thus, under the simulated compression condition of 100 m/s, increasing relative density alone did not produce a corresponding improvement in SEA of the ABS gyroid structures. Their design should therefore jointly consider load-bearing capacity, crushing stability, effective crushing stroke, and mass efficiency.

## Figures and Tables

**Table 1:** C values corresponding to six relative densities of the gyroid structure.

Relative Density (%)	20	25	30	35	40	45
*C*	0.311	0.388	0.465	0.541	0.617	0.692

**Table 2:** Experimental matrix.

Sample ID	Relative Density	Velocity	Sample ID	Relative Density	Velocity
RD0.3–QS–sample1	30%	6 mm/min	RD0.4–QS–sample1	40%	6 mm/min
RD0.3–QS–sample2	30%	6 mm/min	RD0.4–QS–sample2	40%	6 mm/min
RD0.3–QS–sample3	30%	6 mm/min	RD0.4–QS–sample3	40%	6 mm/min
RD0.3–0.1 m/s–sample1	30%	0.1 m/s	RD0.4–0.1 m/s–sample1	40%	0.1 m/s
RD0.3–0.1 m/s–sample2	30%	0.1 m/s	RD0.4–0.1 m/s–sample2	40%	0.1 m/s
RD0.3–0.1 m/s–sample3	30%	0.1 m/s	RD0.4–0.1 m/s–sample3	40%	0.1 m/s
RD0.3–1 m/s–sample1	30%	1 m/s	RD0.4–1 m/s–sample1	40%	1 m/s
RD0.3–1 m/s–sample2	30%	1 m/s	RD0.4–1 m/s–sample2	40%	1 m/s
RD0.3–1 m/s–sample3	30%	1 m/s	RD0.4–1 m/s–sample3	40%	1 m/s
RD0.3–10 m/s–sample1	30%	10 m/s	RD0.4–10 m/s–sample1	40%	10 m/s
RD0.3–10 m/s–sample2	30%	10 m/s	RD0.4–10 m/s–sample2	40%	10 m/s
RD0.3–10 m/s–sample3	30%	10 m/s	RD0.4–10 m/–sample3	40%	10 m/s

**Table 3:** Basic material properties of ABS.

Parameters	Density (kg/m^3^)	Young’s Modulus (GPa)	Poisson’s Ratio	Yield Stress (MPa)
values	1050	1.17	0.3	38.34

**Table 4:** True stress–plastic strain data at different strain rates used as input to the material model.

Q-S		1 s^−1^		100 s^−1^	
$\varepsilon_{p}^{\mathrm{true}}$	*σ_true_* (MPa)	$\varepsilon_{p}^{\mathrm{true}}$	*σ_true_* (MPa)	$\varepsilon_{p}^{\mathrm{true}}$	*σ_true_* (MPa)
0	34	0	51.54	0	59.81
0.01	34.002	0.01	51.59	0.01	59.813
0.02	34.005	0.02	51.6	0.02	59.818
0.03	34.006	0.03	51.604	0.03	59.82
0.04	34.01	0.04	51.61	0.04	59.827
0.05	34.013	0.05	51.617	0.05	59.831
0.06	34.018	0.06	51.62	0.06	59.837
0.07	34.02	0.07	51.625	0.07	59.84
0.08	34.026	0.08	51.63	0.08	59.848
0.09	34.03	0.09	51.65	0.09	59.853
0.1	34.09	0.1	51.68	0.1	59.862
0.11	34.16	0.11	51.71	0.11	59.87
0.12	34.23	0.12	51.73	0.12	60.01
0.13	34.3	0.13	51.76	0.13	60.15
0.14	34.42	0.14	51.79	0.14	60.33
0.15	34.55	0.15	51.82	0.15	60.53
0.16	34.69	0.16	51.85	0.16	60.73
0.17	34.83	0.17	51.88	0.162	62.04
0.18	34.97	0.176	51.89		
0.182	34.98				

**Table 5:** Element counts for gyroid models with different relative densities.

Structure Type	RD 0.2	RD 0.25	RD 0.3	RD 0.35	RD 0.4	RD 0.45
Number of elements	2,025,821	2,030,391	2,363,700	2,536,741	2,644,285	3,001,596

**Table 6:** Statistical results of repeated compression tests.

Relative Density	Velocity (m/s)	IPCF (kN)	MCF (kN)	CFE	EA (J)	SEA (J/g)
30%	0.0001	10.82 ± 0.20	9.65 ± 0.03	0.89 ± 0.01	241.33 ± 0.74	6.13 ± 0.02
	0.1	15.25 ± 0.25	13.05 ± 0.36	0.86 ± 0.02	326.34 ± 8.90	8.29 ± 0.23
	1	16.75 ± 0.26	14.12 ± 0.53	0.83 ± 0.01	348.02 ±10.90	8.84 ± 0.28
	10	17.67 ± 0.2	14.36 ± 0.54	0.81 ± 0.03	355.66 ± 7.41	9.03 ± 0.19
40%	0.0001	15.56 ± 0.75	14.09 ± 0.42	0.91 ± 0.02	352.43 ±10.45	6.71 ± 0.20
	0.1	22.10 ± 0.76	19.72 ± 0.42	0.89 ± 0.01	493.07 ±10.38	9.39 ± 0.20
	1	23.76 ± 0.20	20.91 ± 0.22	0.88 ± 0.01	522.72 ± 5.58	9.96 ± 0.11
	10	26.30 ± 1.07	22.29 ± 0.76	0.85 ± 0.01	556.06 ±11.99	10.59 ± 0.23

**Table 7:** Experimental and simulated IPCF values and relative errors.

Relative Density	Compression Velocity (m/s)	IPCFexp (kN)	IPCFsim (kN)	Error (%)
30%	0.0001	10.82	11.98	10.72
	0.1	15.25	15.66	2.69
	1	16.75	16.7	0.30
	10	17.67	18.99	7.47
40%	0.0001	15.56	17.69	13.69
	0.1	22.1	21.8	1.36
	1	23.76	24.64	3.70
	10	26.30	28.28	7.53

**Table 8:** Experimental and simulated MCF values and relative errors.

Relative Density	Compression Velocity (m/s)	MCFexp (kN)	MCFsim (kN)	Error (%)
30%	0.0001	9.65	10.51	8.90
	0.1	13.05	13.42	2.82
	1	14.09	13.74	2.48
	10	14.36	15.49	7.90
40%	0.0001	14.09	15.74	11.69
	0.1	19.72	19.53	0.97
	1	20.91	21.76	4.07
	10	22.29	25.45	14.16

**Table 9:** Experimental and simulated CFE values and relative errors.

Relative Density	Compression Velocity (m/s)	CFEexp	CFEsim	Error (%)
30%	0.0001	0.89	0.88	1.74
	0.1	0.86	0.87	1.63
	1	0.83	0.86	3.30
	10	0.81	0.84	3.19
40%	0.0001	0.91	0.89	1.83
	0.1	0.89	0.88	1.86
	1	0.88	0.87	0.79
	10	0.85	0.87	2.61

**Table 10:** Experimental and simulated EA values and relative errors.

Relative Density	Compression Velocity (m/s)	EAexp (J)	EAsim (J)	Error (%)
30%	0.0001	241.33	251.84	4.36
	0.1	326.34	327.92	0.48
	1	348.02	341.21	1.96
	10	355.66	389.71	9.57
40%	0.0001	352.43	379.21	7.60
	0.1	493.07	467.03	5.28
	1	522.72	521.01	0.33
	10	556.06	595.58	7.11

**Table 11:** Experimental and simulated SEA values and relative errors.

Relative Density	Compression Velocity (m/s)	SEAexp (J/g)	SEAsim (J/g)	Error (%)
30%	0.0001	6.13	6.40	4.36
	0.1	8.29	8.33	0.48
	1	8.84	8.67	1.96
	10	9.03	9.90	9.57
40%	0.0001	6.71	7.22	7.60
	0.1	9.39	8.90	5.28
	1	9.96	9.92	0.33
	10	10.59	11.34	7.11

## Data Availability

The original contributions presented in this study are included in the article. Further inquiries can be directed to the corresponding author.
